# Clinical Research Progress of Small Molecule Compounds Targeting Nrf2 for Treating Inflammation-Related Diseases

**DOI:** 10.3390/antiox11081564

**Published:** 2022-08-12

**Authors:** Zhenzhen Zhai, Yanxin Huang, Yawei Zhang, Lili Zhao, Wen Li

**Affiliations:** 1 School of Pharmaceutical Sciences, Zhengzhou University, Zhengzhou 450001, China; 2 Collaborative Innovation Center of New Drug Research and Safety Evaluation, Henan Province & Key Laboratory of Advanced Drug Preparation Technologies, Ministry of Education & Key Laboratory of Henan Province for Drug Quality and Evaluation, Zhengzhou 450001, China

**Keywords:** Nrf2, clinical research, small molecule compounds, inflammation-related diseases, COVID-19, Alzheimer’s disease, Parkinson’s disease, lupus erythematosus, liver damage

## Abstract

Studies have found that inflammation is a symptom of various diseases, such as coronavirus disease 2019 (COVID-19) and rheumatoid arthritis (RA); it is also the source of other diseases, such as Alzheimer’s disease (AD), Parkinson’s disease (PD), lupus erythematosus (LE), and liver damage. Nrf2 (nuclear factor erythroid 2-related factor 2) is an important multifunctional transcription factor in cells and plays a central regulatory role in cellular defense mechanisms. In recent years, several studies have found a strong association between the activation of Nrf2 and the fight against inflammation-related diseases. A number of small molecule compounds targeting Nrf2 have entered clinical research. This article reviews the research status of small molecule compounds that are in clinical trials for the treatment of COVID-19, rheumatoid arthritis, Alzheimer’s disease, Parkinson’s disease, lupus erythematosus, and liver injury.

## 1. Introduction to Nrf2 Function

**Figure 1:**
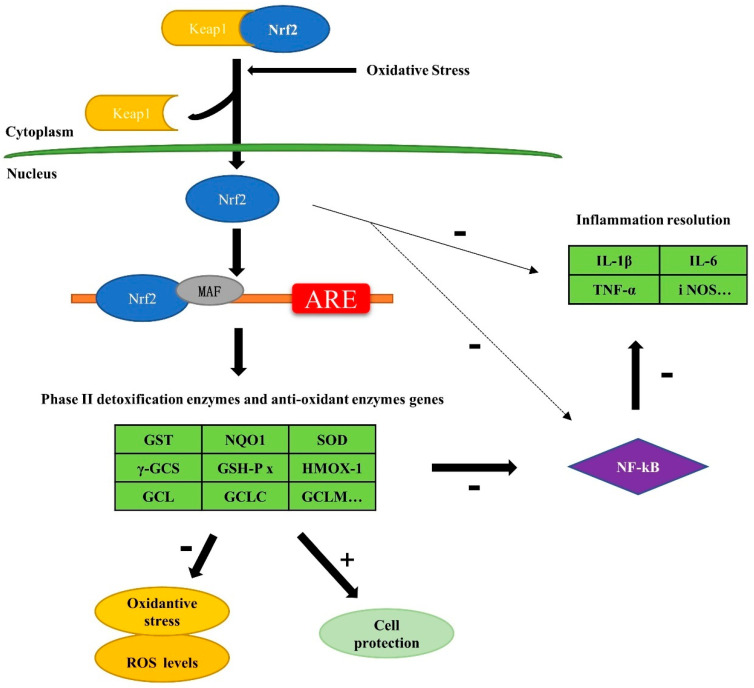
Mechanisms of Nrf2 signaling pathway regulating inflammation.

Oxidative stress refers to the imbalance between oxidation and antioxidants and is caused by the production of reactive oxygen species (ROS) in the body, resulting in oxidative damage to tissue and cells. The Nrf2/Keap1 pathway is the principal protective response to oxidative and electrophilic stresses. Kelch-like ECH-associated protein 1 (Keap1) is a component of the Cullin 3 (CUL3)-based E3 ubiquitin ligase complex and controls the stability and accumulation of Nrf2 [1,2,3,4,5,6,7]. Normally, Nrf2 exists in the cytoplasm under the regulation of Keap1 and maintains low activity in a normal physiological state. When cells are stimulated by oxidative stress, Nrf2 detaches from Keap1 and translocates into the nucleus to form heterodimers with musculoaponeurotic fibrosarcoma (MAF), bind antioxidant response element (ARE), and activate the expression of Nrf2 target genes (Phase II detoxification enzymes and antioxidant enzyme genes), such as heme-oxigenase-1(HMOX-1), NAD (P) H-quinone oxidoreductase 1 (NQO1) and glutamate cysteine ligase (GCL), glutathione S-transferase(GST), superoxide dismutase (SOD), γ-glutamyl cysteine synthetase (γ-GCS), glutathione peroxidase (GSH-Px), γ-glutamyl cysteine synthetase catalytic subunit(GCLC), γ-glutamyl cysteine synthetase modifier subunit(GCLM), etc. [8,9]. The functions of the proteins they encode are as follows: HO-1 is encoded by the HMOX-1 gene, which catalyzes the decomposition of heme with cytochrome P450 to produce biliverdin, etc., and then biliverdin is converted into bilirubin. Both biliverdin and bilirubin have antioxidant and immunomodulatory properties [10]. NQO1 protects cells from the harmful effects of quinone redox cycling [11]. GCL consists of GCLC and GCLM and is the rate-limiting enzyme in the glutathione biosynthetic pathway. GST mainly catalyzes the covalent combination of various chemicals and their metabolites with the sulfhydryl group of glutathione (GSH), making electrophilic compounds into hydrophilic substances, which are easy to excrete [12,13]. SOD catalytically converts the superoxide radical to hydrogen peroxide (H_2_O_2_), constituting the first line of defense against oxidative stress [14]. γ-GCS catalyzes the rate-limiting biosynthesis of GSH, an abundant physiological antioxidant that plays important roles in regulating oxidative stress. GSH-Px specifically catalyzes the reaction of GSH with ROS, thereby protecting cells from ROS damage [15,16]. Nuclear factor kappa B (NF-kB) is closely related to the regulation of inflammation by participating in the activation of genes encoding proinflammatory cytokines, growth factors, and inducible enzymes, such as interleukin 1 beta (IL-1β), interleukin 6 (IL-6), tumor necrosis factor alpha (TNF-α), and inducible nitric oxide synthase (iNOS). Nrf2 reduces inflammatory response by inhibiting the activity of NF-kB through the Nrf2-ARE pathway and by directly inhibiting the activity of NF-kB and the expression of proinflammatory cytokine genes (Figure 1) [17]. Numerous studies have shown that Nrf2 and NF-kB play important roles in regulating cancer responses to chemotherapy [18,19] and the immune/inflammatory cancer microenvironment in almost all types of cancer [20].

## 2. Research Progress of Nrf2 in Inflammation-Related Diseases

### 2.1. Nrf2 and Coronavirus Disease 2019 (COVID-19)

**Figure 2:**
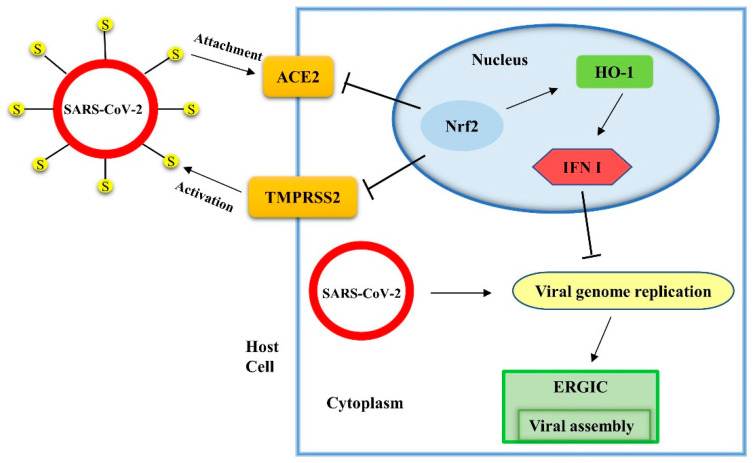
Antiviral effects of Nrf2 pathway on SARS-CoV-2.

COVID-19 is a complex infectious disease caused by severe acute respiratory syndrome coronavirus 2 (SARS-CoV-2). Clinical evidence has shown that the main symptoms of COVID-19 can include acute infection of the respiratory tract, as well as inflammatory reactions of multiple organs [21,22]. SARS-CoV-2 enters cells by first binding to angiotensin-converting enzyme 2 (ACE2), followed by cleavage of the virus spike protein by transmembrane protease serine 2 (TMPRSS2) [23]. Nrf2 located in the nucleus can directly inhibit the expression of ACE2 and TMPRSS2 on the cell surface to reduce SARS-CoV-2 entry into cells; it can also block the replication of the viral genome by mediating the production of type I interferons (IFN-I) by HO-1 [24,25,26]. Through these two mechanisms, the Nrf2 signaling pathway can effectively reduce SARS-CoV-2 infection (Figure 2).

**Scheme 1:**
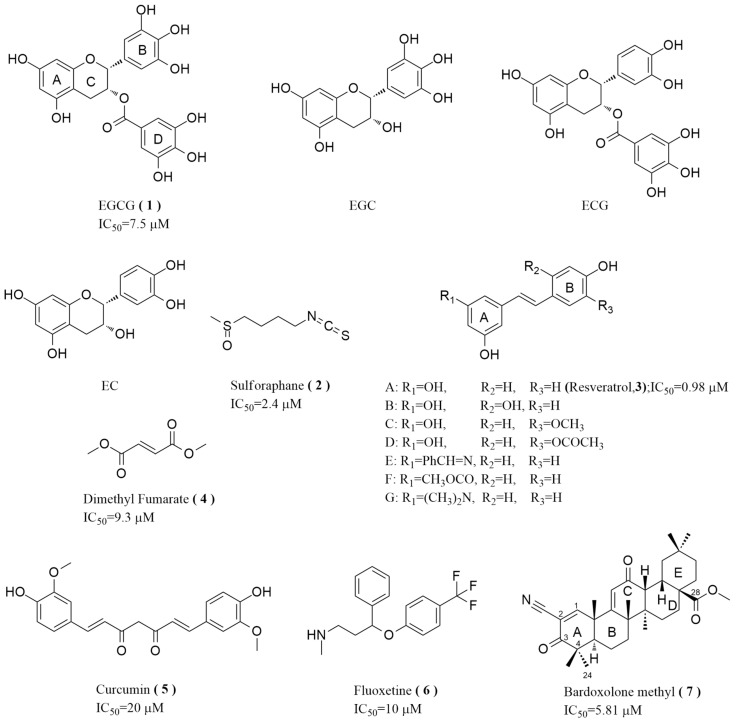
Structures and numbers of seven Nrf2 agonists promising for the treatment of COVID-19.

At present, seven Nrf2 agonists have entered clinical trials as COVID-19 treatments (Scheme 1 and Table 1). These compounds are discussed below.

Epigallocatechin gallate (EGCG) (**1**, 50% inhibitory concentration (IC_50_) is 7.51 μM [27]) (3CL Protease (M^pro^) (SARS-CoV-2) Assay Kit [BPS bioscience, https://bpsbioscience.com/ (accessed on 3 August 2022)] using the fluorescence method) is the main component of green tea polyphenols; it is a catechin monomer isolated from tea and is a flavanol compound. EGCG has entered phase 2/3 clinical trials. According to the strength of oxidation, the order of oxidation of EGCG and its three derivatives ((Epigalloeatechin)EGC, (Epieatechin gallate) ECG, (Epieatechin) EC) is: EGCG > EGC > ECG > EC [27,28,29,30,31,32,33,34,35]. Therefore, it can be speculated that the 3′,4′,5′-trihydroxyl group of the B ring in the EGCG structure is important for its antioxidant capacity. The gallic acid ester of the D ring also contributes to its antioxidant capacity, and studies have shown that the two may be involved in metal chelation. Multiple phenolic hydroxyl groups endow EGCG with robust antioxidant activity, high hydrophilicity, and active properties, so its stability should be fully considered when designing a drug.

Sulforaphane (**2**, IC_50_ is 2.4 μM ) (quantification of viral RNA from SARS-CoV-2-infected human intestinal Caco-2 cells treated with SFN using qPCR) is produced through the hydrolysis of glucosinolates, which are found in cruciferous vegetables, such as cabbage and radishes [36,37,38,39,40,41,42,43]. Sulforaphane has entered phase 2 clinical trials. The bioavailability of sulforaphane is approximately 80%. However, its disadvantages are its instigation of strong irritation, volatility, and sensitivity to temperature and pH. The isothiocyanate group is the pharmacophore of sulforaphane. Converting methylsulfinyl to an acetyl group or N-methylformamide reduces its antioxidant capacity; however, when converted to squaramide, its antioxidant capacity is 25 times greater than that of sulforaphane [44]. Therefore, it is possible to modify the structure of the butyl carbon chain of sulforaphane to make it less irritating and more stable.

Resveratrol (**3**, IC_50_ is 0.98 μM [45]) (detection of the inhibitory activity of resveratrol on COX-2 enzymes using a COX-2 inhibitor screening kit using the fluorescence method) is found in plants such as blackberries, peanuts, and grapes and has good anti-inflammatory and anti-SARS-CoV-2 activities as an Nrf2 agonist [45,46,47,48,49]; this compound has entered phase 3 clinical trials. The resveratrol–ibuprofen combination, in which the hydroxyl group on the B ring is monosubstituted, has a more significant anti-inflammatory effect than either compound given alone [50]. Pei Ling et al. once discussed the relationship between antioxidation and chemical structure of resveratrol and its analogues (Scheme 1) and put forward the concept of hydrogen-donating ability. Using quantum chemical calculations based on the density functional theory (DFT), they calculated the hydrogen-donating ability of these compounds. The order of these compounds is C > D > B > G > E > A > F, and the antioxidation of these compounds is positively correlated with their hydrogen-donating ability [51].

Dimethyl fumarate (DMF) (**4**, IC_50_ is 9.30 μM [52]) (detection of the inhibitory activity of DMF on IL-6 produced by lipopolysaccharide-induced dTHP-1 cell line using IL-6 commercial kits (Perkin Elmer) and the fluorescence method) is a US Food and Drug Administration (FDA)-approved synthetic drug used as an anti-inflammatory therapeutic for multiple sclerosis (MS) via Nrf2 inhibition of pathogenic inflammation [53,54], and it is currently in phase 2/3 clinical trials. Isosorbide di-(methyl fumarate) (IDMF), with a central isosorbide moiety and two methyl fumarate groups, can partially replicate DMF activity and is nonirritating and nonsensitizing when applied to the skin [55]. When the carboxyl groups at both ends of the DMF structure were changed to 4-chlorophenyl ester, Ar-NH-, and Ar-CH2-NH-, anti-inflammatory efficacy was greatly improved [56]. Based on the above, we preliminarily hypothesize that the intermediate chain ketone-ene-ketone structure of DMF may be an essential group for its activity and that the carboxyl groups at both ends can be transformed to synergistically improve the detoxification of the compound.

Curcumin (**5**, IC_50_ is 20 μM [57]) (Researchers pulsed bone-marrow-derived dendritic cells (BMDCs) for 1 h with curcumin before stimulation with the TLR7 ligand R837 followed by ATP to investigate IL-1β production.) is a diketone compound extracted from the rhizomes of plants in the Zingiberaceae and Araceae families [54,58,59]. It is in phase 4 clinical trials. The unsaturated carbon chain and hydroxyl group on the benzene ring of curcumin are extremely important for its anti-inflammatory activity. The alkoxy group next to the phenol group and the benzene ring substituted by the strong electron withdrawing group of the ortho-diphenol hydroxyl group can increase its anti-inflammatory ability [60,61]. The hydrophobicity and rapid metabolism of curcumin lead to poor bioavailability. Some studies have structurally modified the phenolic hydroxyl groups at both ends to transform them into ether, which effectively slowed the metabolism of the compound [62].

Fluoxetine (**6**, IC_50_ is 10 μM [63]) (Different concentrations of fluoxetine were added to Vero E6 cell cultures along with SARS-CoV-2, and the levels of infectious particles in culture supernatants were detected by incubation.) is a synthetic drug that was first approved in Belgium in 1986 for the treatment of depression. It is one of the few classic clinical drugs with Nrf2 agonistic effects [63,64,65,66]. It has entered phase 3 clinical trials. There is almost no anti-inflammatory activity when the methylamino group in fluoxetine is replaced by pyrrolidine, imidazole or piperidine, but there is equivalent activity when replaced by morpholine, piperazine, or N-methylpiperazine [67]. In the case of removing the trifluoromethyl benzene ring and replacing the methylamino group with morpholine, there is no anti-inflammatory activity when the ether bond is replaced by a hydroxyl group and an oxime group; when it is replaced by a ketone group, the activity is comparable to that of fluoxetine. There is increased anti-inflammatory activity after the introduction of trifluoromethyl to the carbon [68]. Therefore, it can be speculated that fluoxetine, methylamino groups, trifluoro-methylbenzene rings, and ether linkage are the key groups that affect the activity of fluoxetine analogues.

Bardoxolone methyl (**7,** IC_50_ is 5.81 μM [69]) (detection of the inhibitory activity of Bardoxolone methyl on SARS-CoV-2 3CLpro with Thr-Ser-Ala-Val Leu-Gln-pNA-substrate by using absorbance at 390 nm) is a semisynthetic pentacyclic triterpenoid derived from oleanolic acid [69,70,71]. It has entered phase 2 clinical trials. Suqing Zheng et al. synthesized a series of monocyclic cyanoketene compounds and tested their anti-inflammatory ability. The study showed that the pharmacophore in semisynthetic pentacyclic triterpenoids is not pentacyclic triterpenoid and has nonenolized cyanoketenes rather than a tricyclic skeleton [72]. It is speculated that the A-rings are necessary for the anti-inflammatory activity of bardoxolone methyl. They function as Michael receptors, and the single-ring structure is more potent than the penta-ring structure. One study showed that removal of C-24 at the C-4 position of the A ring led to higher biological activity and that transforming methyl 28-carboxylate into ethylamide or trifluoroethylamide improved drug delivery to the brain [73,74]. Thus, the structural modification of C-28 is expected to alter its pharmacokinetic properties.

### 2.2. Nrf2 and Rheumatoid Arthritis

**Figure 3:**
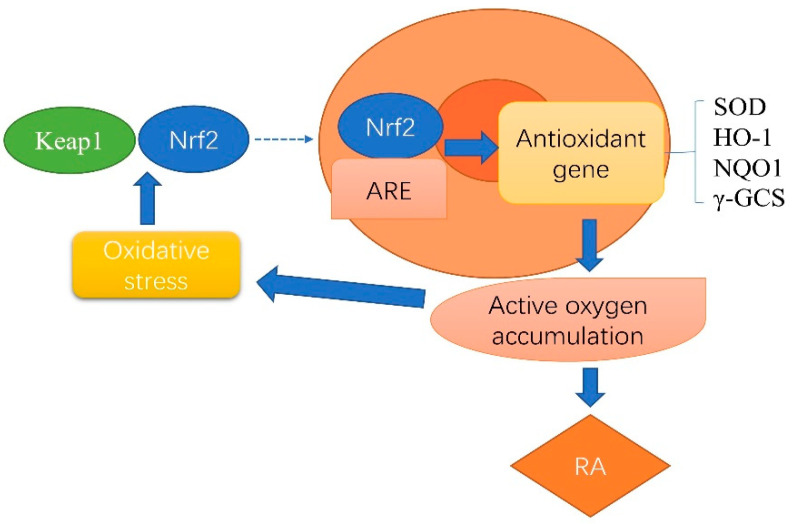
Mechanism of Keap1-Nrf2/ARE signaling pathway in RA.

Rheumatoid arthritis (RA) is a chronic autoimmune disease of unknown etiology and affects approximately 0.5–1.0% of the world’s population. It often presents with joint involvement, synovitis, and intra-articular cartilage damage [75,76]. It is thought that the etiology of RA is closely related to one’s living environment, genetics, immunity, and other factors. Individuals with genetic factors are affected by their living environment, stress, and other factors, which induce abnormal responses in the innate and adaptive immune systems, leading to the destruction of immune tolerance and thus stimulating an inflammatory response [77,78]. The main pathological feature of RA is inflammation leading to articular cartilage damage caused by cartilage degradation. Many studies have shown that Nrf2 activation is a promising method for the treatment of RA [79]. The Kelch-Nrf2/ARE signal transduction pathway can have beneficial anti-inflammatory and antioxidant effects and can regulate oxidative stress in RA. At its core, increased Nrf2 activity can regulate mitochondrial function and limit the production of mitochondrial ROS after activation of this pathway [80] (Figure 3).

At present, two Nrf2 agonists have entered clinical research for rheumatoid arthritis (Scheme 1 and Table 2).

First, research has shown that 10 μM (**4**) significantly inhibits the formation and activity of osteoclasts, and the excessive formation of osteoclasts is related to the bone destruction pathology seen in RA [81,82,83,84,85,86]. Compound (**4**) has entered phase 2 clinical trials. Second, 50 μM (**5**) significantly inhibited the activity of collagen-induced arthritis (CIA) in mouse cells and the expression of proinflammatory factors. These results point to the anti-RA effect [87,88,89,90,91,92] of (**5**), which has entered phase 1 clinical trials.

### 2.3. Nrf2 and Alzheimer’s Disease

**Figure 4:**
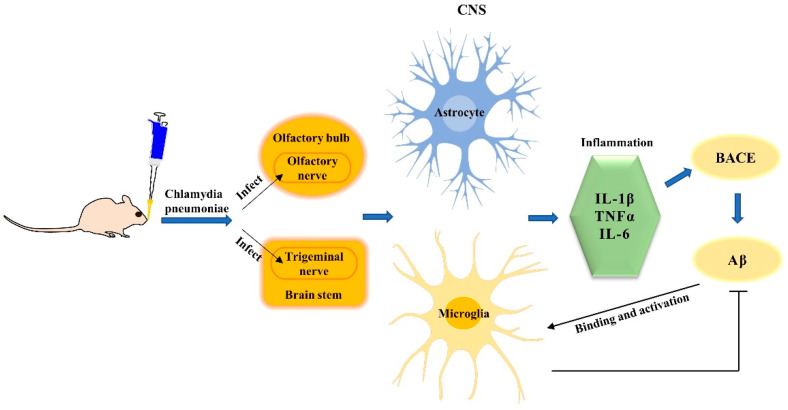
*Chlamydia pneumoniae* infection contributes to the pathogenesis of Alzheimer’s disease.

Senile plaques formed through the accumulation of β-amyloid(Aβ) and neurofibrillary tangles caused by hyperphosphorylation of tau protein are important pathological features of AD [93]. AD affects more than 50 million people. There are various pathogenic hypotheses of AD, such as the cholinergic hypothesis, the Aβ toxicity hypothesis, the tau protein hypothesis, and the inflammation hypothesis, but the pathogenesis of AD still must be elucidated [94]. A recent experiment showed that *Chlamydia pneumoniae* infection is closely related to AD pathogenesis. *Chlamydia pneumoniae* was shown to enter the nasal cavity of mice and rapidly infect the olfactory and trigeminal nerves, which connect to the brain through the olfactory bulb and brain stem, respectively. Microglia and astrocytes (macrophages of the central nervous system (CNS)) can respond to and engulf bacteria. However, *Chlamydia pneumoniae* can evade destruction by phagocytes and infect glial cells by forming inclusion bodies in these cells. Following infection, activated microglia and astrocytes secrete proinflammatory cytokines, including IL-1β, TNFα, and IL-6, which are neurotoxic and directly increase Aβ production by activating β-site amyloid-precursor-cleaving enzyme (BACE). On one hand, activated microglia reduce the accumulation of Aβ in the brain by increasing their phagocytosis, clearance, and degradation. On the other hand, the continuous activation of microglia caused by their binding to Aβ can increase the production of inflammatory mediators, which further amplifies the neuroinflammatory response, leading to chronic inflammation and AD [95,96,97,98] (Figure 4).

In animal models of AD, Nrf2 inhibits its expression by binding to AREs in the BACE promoter and inhibits Aβ production. It can also induce nuclear dot protein 52 (NDP52) by binding to AREs in the NDP52 promoter, thereby reducing p-tau levels in AD [99,100,101]. Therefore, the activation of Nrf2 by drug intervention may play a positive role in treating AD patients.

Currently, four Nrf2 agonists have entered clinical research related to AD treatment (Scheme 1 and Table 3). Compound (**1**) reduces the production of Aβ through the Nrf2 pathway and directly binds to Aβ monomers and dimers, leading to structural remodeling of Aβ and reducing its toxicity [102,103,104,105,106,107]. This compound has entered phase 2/3 clinical trials. Compound (**2**) effectively inhibits the production of inflammatory mediators in microglia and improves memory deficits [108,109,110,111]. The clinical trial status of (**2**) has not yet been announced. Compound (**3**) can reduce neuronal oxidative damage [112,113] and has entered phase 3 clinical trials. Compound (**5**) reduces Aβ-induced cell death and oxidative stress and significantly improves spatial memory deficits in AD mice [114,115,116] and has entered phase 2 clinical trials.

### 2.4. Nrf2 and Parkinson’s Disease

**Figure 5:**
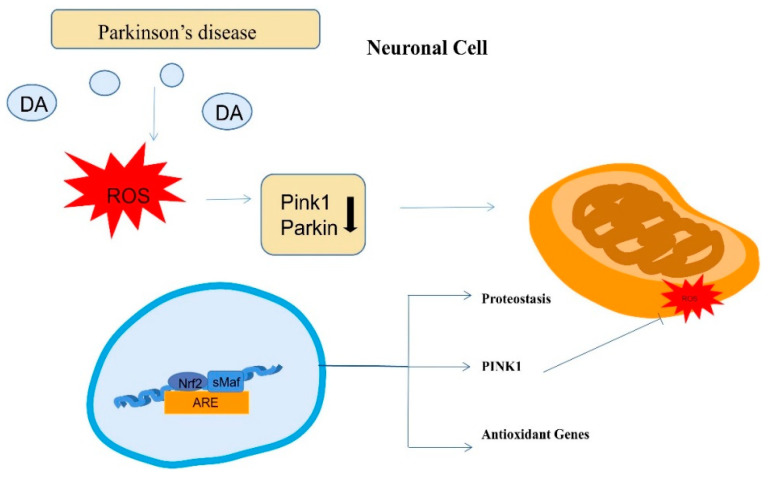
The mechanism of Nrf2 in PD.

Parkinson’s disease (PD) is a chronic progressive nervous system disease. In late-stage PD, extreme tremors, motor retardation, muscle stiffness, and loss of balance occur [117]. In sporadic and familial PD, α-synuclein(α-syn) aggregates into Lewy bodies and Lewy neurites, which are cytotoxic to dopaminergic neurons and can lead to mitosis and enhance mitochondrial autophagy [118]. The increase in dopamine may affect mitochondrial function, increase ROS levels, affect Nrf2 activity, alter the response to antioxidant damage [119,120,121], and promote the progressive production and accumulation of Aβ [122]. These effects lead to dysregulated cellular function. However, Nrf2 activation can neutralize ROS, inhibit inflammatory processes, and restore cellular redox balance [123,124,125,126,127]. In PD, there are decreased protein expression levels of phosphatase and tensin homolog (PTEN)-induced kinase (PINK) and Parkin protein; the decreases in these proteins affect mitochondrial function, induce depolarization and fragmentation and reduce adenosine triphosphate (ATP) concentrations (Figure 5) [128]. These changes will affect synaptic function, leading to neurodegeneration and cognitive impairment [124,125,126,127].The Nrf2 upregulation induced by antioxidant therapy was shown to enhance thioredoxin-1(TrX-1), inhibit the formation of nucleotide-binding domain leucine-rich repeat-related (NLR) family pyrin domain-containing 3 (NLRP3) inflammatory bodies and improve neuronal apoptosis in amyloid precursor protein plus presenilin-1 (APP/PS1) mice [129]. Although some mechanisms are not fully understood, Nrf2 can be considered a useful therapeutic target for PD [130].

**Scheme 2:**
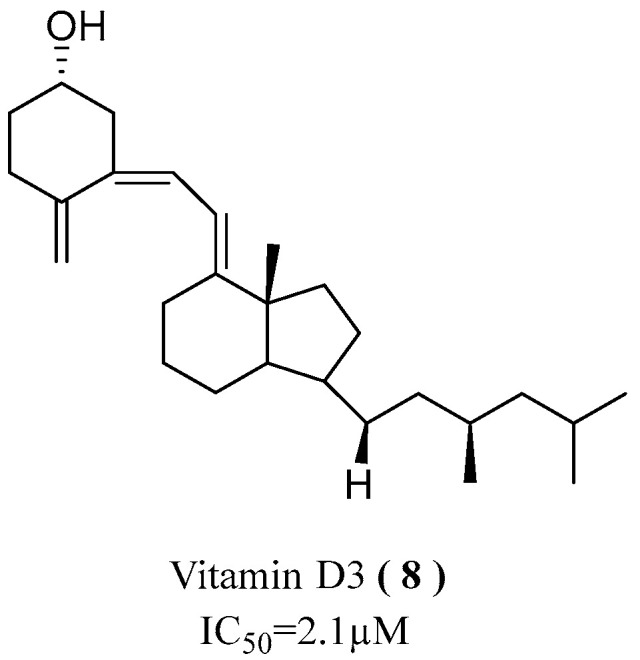
Structures of Vitamin D3.

Four Nrf2 agonists have entered clinical trials for the treatment of PD (Scheme 1 and Scheme 2 and Table 4).

Vitamin D3 (**8**, IC_50_ is 2.1 µM [131]) (the ability of VD3 in C3H10T1/2 fibroblasts to down-regulate gli1mrna expression in a dose-dependent manner) is an important regulator of bone metabolism and calcium and phosphorus balance. It is converted from 1α-hydroxylase to its active metabolite 1,25(OH)2D and is currently in phase 4 clinical trials [132,133]. The 1-hydroxy group and the 10-position exomethylene group play important roles in maintaining the activity of the compound. Most research has focused on the modification of side chains and the A ring [134]. Compound (**2**) can cross the blood–brain barrier. The treatment with 0.1% glucoraphanin pellets preserved dopaminergic neurons from neurodegeneration [135,136]. Currently, in phase 2 clinical trials, Compound (**1**) (20 µmol/L) acts by upregulating antioxidase activity [137], and it can effectively scavenge H2O2 [138]. EGCG is currently in phase 2 clinical trials for the treatment of PD. Monoamine oxidase (MAO) regulates the local levels of neurotransmitters such as dopamine, norepinephrine and serotonin, and (3) has a selective inhibitory effect on MAO-A [139]. Compound (**3**) is currently in phase 1 clinical trials.

### 2.5. Nrf2 and Lupus Erythematosus

Systemic lupus erythematosus (SLE) is a chronic disease characterized by the loss of immune tolerance. SLE has a variety of clinical manifestations, the main sign of which is the production of autoantibodies that cause tissue damage [140]. Toll-like receptor 9 (TLR9) is an important bridge linking innate and adaptive immunity. For example, when the body is subjected to specific external stimuli, TLR9 activates the NF-κB pathway, leading to inflammation. T helper type 17 (Th17) cells are major proinflammatory T cells involved in the regulation of lupus nephritis (LN) through multiple mechanisms.

**Figure 6:**
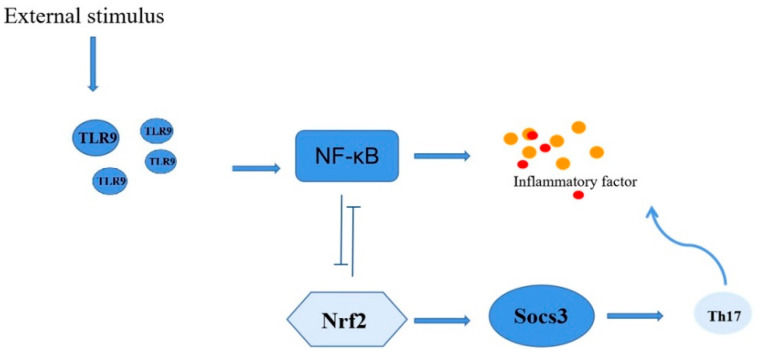
The mechanism of Nrf2 in SLE.

Signal transducer and activator of transcription 3 (STAT3) directly regulates interleukin-17 (IL-17) expression and suppresses cytokine signaling 3 (Socs3), which negatively regulates Th17 differentiation by downregulating STAT3 phosphorylation. Nrf2 inhibits Th17 differentiation and reduces STAT3 phosphorylation by upregulating Socs3 expression (Figure 6) [141]. SLE can affect bone metabolism and serum electrolysis through renal impairment and by disturbing endocrine homeostasis [142]. In general, dysimmunity, oxidative stress, and inflammation are the key pathogenic features of SLE and LN [143,144]. Preventing SLE development in humans might be facilitated by activating the Nrf2 pathway and applying other antioxidant therapies.

**Scheme 3:**
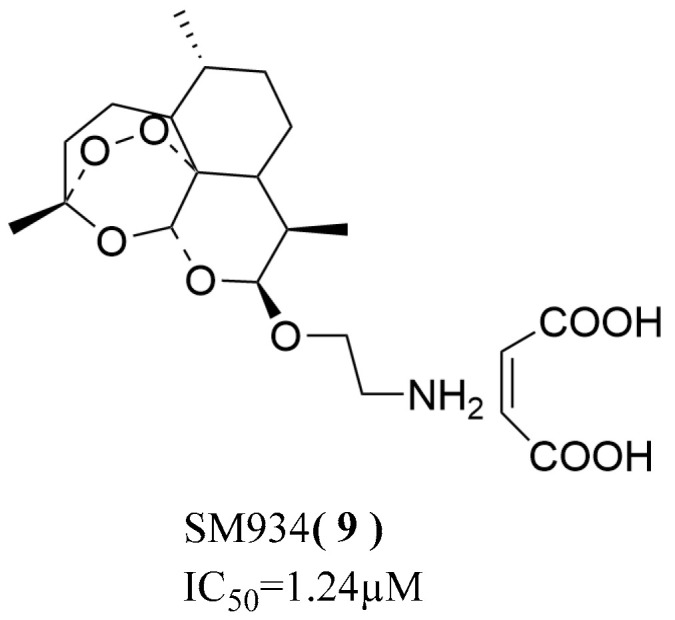
Structure of SM934.

Three Nrf2 agonists have entered clinical research trials as a treatment for SLE (Scheme 1, Scheme 2 and Scheme 3 and Table 5).

Compound (**5**) can inhibit inflammatory pathways, neutralize free radicals, and inhibit ROS production [145,146]. It is currently in phase 2 clinical trials as an immunomodulator for the treatment of SLE. Clinical trials of **(8)** for the treatment of SLE are in phase 2.

β-Aminoarteether maleate (SM934) (**9**, IC_50_ is 1.24 µM [147]) (immunosuppression method of spleen cell proliferation induced by Con and LPS) is a water-soluble derivative of artemisinin. SM934 can inhibit TLR7/9 expression [148,149], renal antibody production, and the accumulation of inflammatory cytokines [150]. This compound has entered phase 2 clinical trials. The peroxyl bridge of SM934 is the key group enabling its functionality. The aminoethyl group in the structure increases its water solubility, reduces toxicity and side effects, and enhances efficacy [151].

### 2.6. Nrf2 and Liver Injury

The liver is the largest digestive gland in the human body. It has basic functions such as secreting bile, breaking down sugars and storing glycogen, detoxification, phagocytosis, and defense. Oxidative stress caused by drugs, viruses, alcohol, and other factors is the main cause of liver damage, which can further aggravate drug-induced liver damage, fatty liver, viral hepatitis, autoimmune liver disease, liver fibrosis, and primary liver cancer. The Nrf2 pathway is widely involved in many aspects of the body’s defense against oxidative stress, such as detoxification, anti-inflammatory processes, and the regulation of cellular metabolism [152,153,154,155,156].

#### 2.6.1. Role of Nrf2 in Nonalcoholic Fatty Liver Disease (NAFLD)

**Figure 7:**
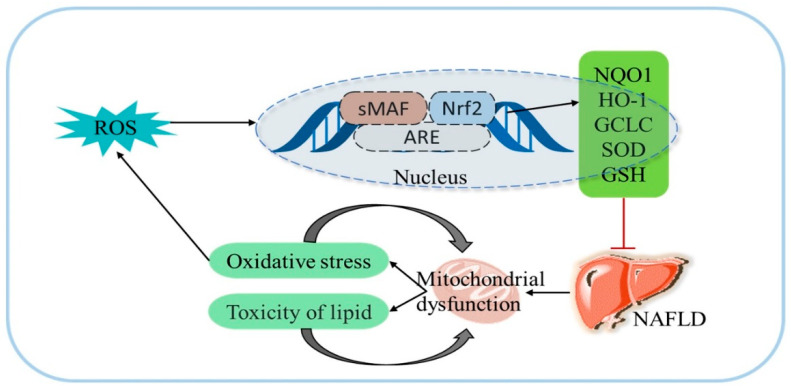
Pathogenesis of NAFLD.

NAFLD is the most common chronic liver disease worldwide and is mainly characterized by a clinicopathological syndrome of excessive deposition of fat in liver cells. It can be caused by excessive alcohol intake and other liver-damaging factors. There are many structural and functional abnormalities in the mitochondria of NAFLD patients [157] that lead to the overproduction of ROS and cytokines. This triggers lipid peroxidation, and the generated ROS and lipid peroxidation products further damage mitochondrial function [158] in a vicious cycle (Figure 7). There is currently no definitive drug treatment for NAFLD.

Three Nrf2 agonists have entered clinical research for the treatment of NAFLD (Scheme 1 and Table 6).

Liraglutide increases the concentrations of Sestrin2 and Nrf2 and improves obesity-related NAFLD [159]. It is currently in phase 4 clinical trials. Resveratrol (**3**) was shown to attenuate methylation of the Nrf2 promoter in the liver of mice fed a high-fat diet (HFD) and attenuated NAFLD through epigenetic modification of Nrf2 signaling [160]. This compound is now in phase 2/3 clinical trials. Curcumin (**5**) treatment significantly alleviated liver steatosis in mice fed an HFD, reversed abnormal serum biochemical parameters, and increased the metabolic capacity to effectively restore the Nrf2-FXR-LXR pathway [161]. Curcumin is currently in phase 2/3 clinical trials.

In addition, a variety of natural Nrf2 activators such as aucubin [162], ginkgolide B [163], and limonin [164] can also alleviate NAFLD by regulating lipid metabolism and oxidative stress in hepatocytes. However, these compounds require further clinical investigation.

#### 2.6.2. Role of Nrf2 in Viral Hepatitis 

**Figure 8:**
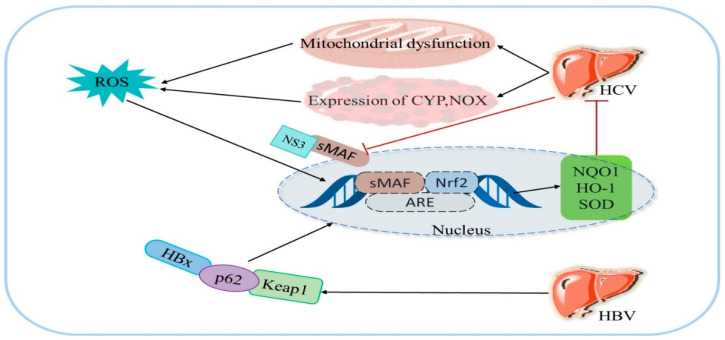
Roles of Nrf2 in viral hepatitis.

The core protein and nonstructural protein 5A (NS5A) of hepatitis C virus (HCV) cause mitochondrial dysfunction in hepatocytes, and the resulting expression of cytochrome P450 2E1 (CYP2E1) and NADPH-oxidase (NOX) produces a large amount of ROS [165,166]. HCV core protein and NS5A can also activate Nrf2 to alleviate HCV [167], while HCV can cause MAF to translocate and bind to extranuclear nonstructural protein 3 (NS3), which then binds to Nrf2 in the cytoplasm, preventing Nrf2 from entering the nucleus [168,169,170]. The hepatitis B x protein (HBx) of hepatitis B virus (HBV) can alter a variety of mitochondria-related functions and is an important cause of mitochondrial dysfunction [171]. HBV can enhance the interaction between p62 and Keap1 to form the HBx-p62-Keap1 complex in the cytoplasm, thereby promoting Nrf2 expression [172] (Figure 8).

**Scheme 4:**
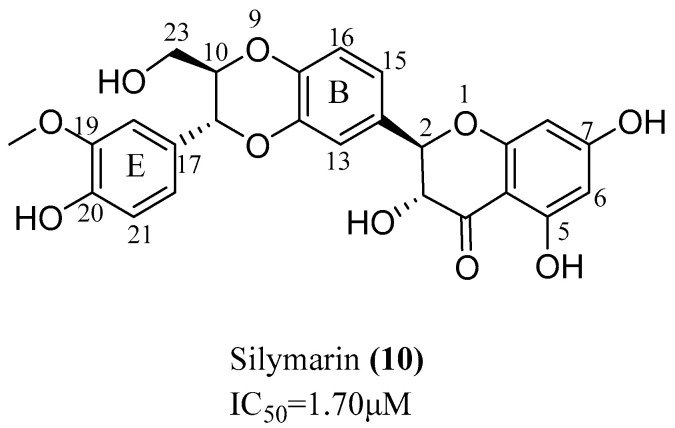
Structures of Silymarin.

Currently, silymarin is the only Nrf2-related compound that has entered clinical trials for the treatment of HCV (Scheme 4 and Table 7). Silymarin (**10**, IC_50_ is 1.70 μM [173]) (measure metabolite concentrations with a fluorescence spectrometer at an excitation wavelength of 409 nm and an emission wavelength of 530 nm; the positive control runs on the same plate), refers to a class of flavonoid lignans extracted from the fruit and seeds of the Compositae herb, *Silybum marianum*; these lignans contain dihydroflavonols and phenylpropanoid derivatives [174]. Silymarin has entered phase 3 clinical trials [175]. Multiple phenolic hydroxyl and methoxy groups endow silymarin with good antioxidant activity. The introduction of methoxy groups on the B ring and the E ring improves the ability of silymarin to scavenge superoxide free radicals and 2,2-diphenyl-1-picrylhydrazyl (DPPH) free radicals [176]. Esterification of the 3- or 23-hydroxyl of silymarin significantly improves its solubility, but its biological activity is reduced [177].

#### 2.6.3. Role of Nrf2 in Primary Biliary Cholangitis

Primary biliary cholangitis (PBC) is an organ-specific chronic and cholestatic autoimmune liver disease. Nrf2 protein concentrations are elevated in PBC patients, but Nrf2 gene expression is significantly decreased, and Keap1 and p62 protein concentrations are significantly increased [178,179]. Aberrant Nrf2/Keap1 system integrity may affect the self-defense mechanism against oxidative stress in PBC.

**Scheme 5:**
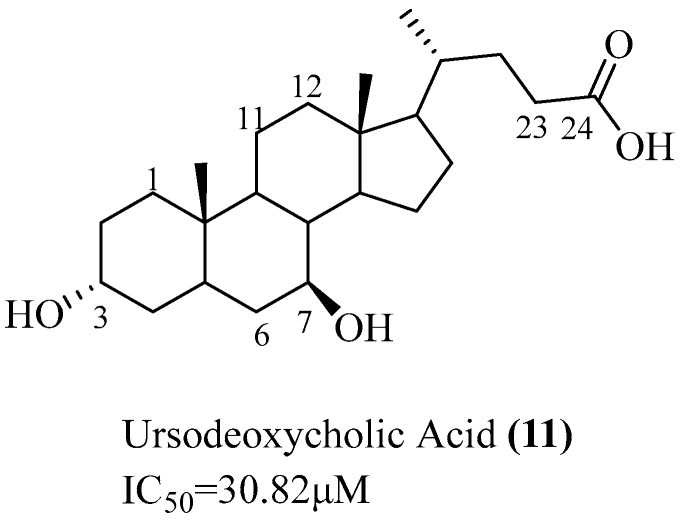
Structures of ursodeoxycholic acid.

Currently, only one Nrf2-related compound has entered clinical trials for PBC (Scheme 5 and Table 8). Ursodeoxycholic acid (**11**, IC_50_ is 30.82 μM [180]) (structure of primary and secondary bile acids as well as corresponding potency in differential scanning fluorimetry binding and cell rounding assays) is a bile acid compound [180]. It is the only drug approved by the US FDA for the treatment of PBC, and it is still the first-line drug for the treatment of PBC. It is currently in phase 4 clinical trials. The 3 and 7 phenolic hydroxyls endow ursodeoxycholic acid with antioxidant activity; this compound also enhances Nrf2 activation in hepatocytes of PBC patients and increases thioredoxin (TRX) and thioredoxin reductase 1 (TrxR1) proteins, thereby relieving PBC [181]. Ursodeoxycholic acid derivatives modified by glycine at position 24 have strong antioxidant effects and fewer toxic side effects than the parent compound [182,183]. The 24-position carboxylic acid is substituted by a heterocycle to obtain a ursodeoxycholic acid derivative that can selectively deliver NO to the liver, significantly increase the concentration of cyclic guanosine 3′, 5′-monophosphate (cGMP) in the liver, and effectively inhibit various inflammatory factors, such as interleukin and tumor necrosis factor [184]. This derivative has a good therapeutic effect for the treatment of liver damage and the associated inflammation.

#### 2.6.4. Role of Nrf2 in Liver Fibrosis

Globally, the number of people with liver fibrosis is expected to increase from 740 million in 2017 to 821 million in 2022. An important cause of liver fibrosis is the activation of hepatic stellate cells (HSC). Ruart et al. found that damage to sinusoidal endothelial cells during acute liver injury aggravates oxidative stress and activates stellate cells to promote liver fibrosis; furthermore, autophagy-impaired liver sinusoidal endothelial cells (LSEC) can cause ROS accumulation and elevated p62 levels, which activates the upregulation of Nrf2 and its target genes [185].

**Scheme 6:**
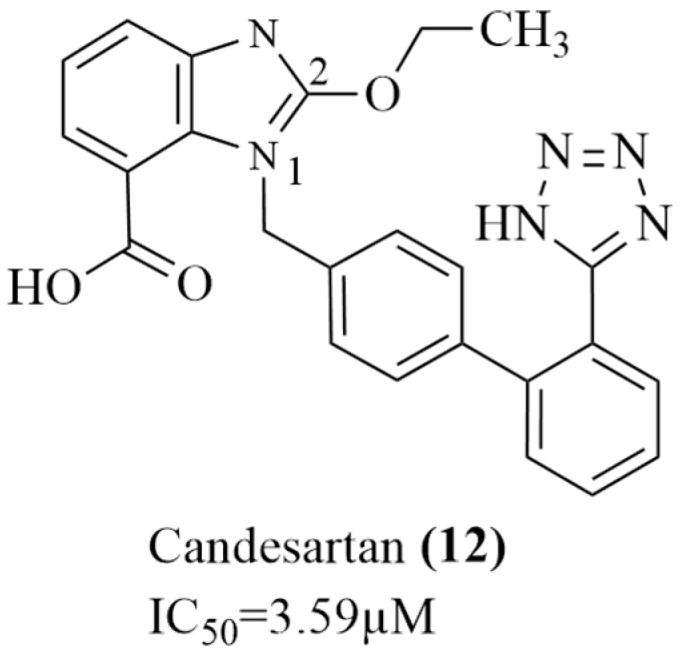
Structures of candesartan.

At present, only one Nrf2-related compound has entered clinical research for the treatment of hepatic fibrosis (Scheme 6 and Table 9). Candesartan (**12**, IC_50_ is 3.59 μM [186]) (Immunofluorescence was conducted with mouse anti-OC43 N protein antibody and followed by Alexa Flour 488 and DAPI. The IC_50_ was calculated using automated image analysis software), is an angiotensin II (Ang II) receptor antagonist. Recent studies have found that candesartan’s antihepatic fibrosis effect occurs partly through the activation of Nrf2 and its downstream target genes [187]. Candesartan is in phase 3 clinical trials. One study found that the substitution of 2-ethoxy increases its antioxidant activity, and the substitution of the carboxyl group at the 4-position of the benzene ring increases its water solubility and improves its pharmacokinetic properties.

In addition, sitagliptin [188,189], liraglutide [190], and mulberrin [191] can reduce aspartate aminotransferase (AST) and alanine aminotransferase (ALT) levels in mouse serum and alleviate stellate cell activation and liver fibrosis. These compounds are currently being investigated.

## 3. Conclusions

In conclusion, 12 of small molecule compounds targeting Nrf2 have entered clinical research for the treatment of inflammation-related diseases. According to different sources, these compounds can be divided into natural products and repurposed drugs.

Compounds **1**, **2**, **3**, **5**, **7**, **9** and **10** are the chemical constituents of natural plants or their structural modifications. Among them, EGCG (**1**) and sulforaphane (**2**) are in clinical trials for the treatment of COVID-19, Alzheimer’s disease, and Parkinson’s disease; resveratrol (**3**) is in clinical trials for the treatment of COVID-19, Alzheimer’s disease, Parkinson’s disease, and liver injury; curcumin (**5**) is in clinical trials for the treatment of COVID-19 and rheumatoid arthritis; oleanolic acid derivatives (**7**) are in clinical trials for the treatment of COVID-19; artemisinin derivative SM934 (**9**) is in clinical trials for the treatment of lupus erythematosus. Silymarin (**10**) is in clinical trials for the treatment of viral hepatitis. 

Compounds **4**, **6**, **8** and **12** are the repurposed drugs. Dimethyl fumarate (**4**) is used in the treatment of multiple sclerosis (MS) in the United States, Europe, and other countries. Now, it is in clinical trials for the treatment of COVID-19 and rheumatoid arthritis; the antidepressant drug fluoxetine (**6**) is in clinical trials for the treatment of COVID-19. Vitamin D3 (**8**) is in clinical trials for the treatment of Parkinson’s disease and lupus erythematosus; the bile acid ursodeoxycholic acid (**11**), a drug used for the treatment of gallstone diseases, is now used in clinical trials for the treatment of autoimmune liver disease. Candesartan (**12**), a lipid-lowering drug, is in the clinical research stage for liver fibrosis.

It should be noted that there is now some genuine structural information about the NRF-2/KEAP system from crystallographic studies carried out in China which identifies a nucleophilic addition of a thiol on the protein target to a Michael acceptor in the drug molecule [192]. Several of the compounds described in this review contain either Michael acceptors or other electrophilic groups to which a thiol would add. Furthermore, the possibility of blocking the NRF-2/KEAP interaction with small molecule drugs has been discussed in some detail by the Strathclyde group led by Harnett [193]. These research results of the specific interaction between the small molecule drugs and target proteins provide a valuable basis for the further design of new drugs targeting Nrf2.

Drugs with various structural types that target Nrf2 have achieved promising clinical experimental results, which confirms the good drug ability of these compounds that target Nrf2. The therapeutic areas involved are diverse, and clinical drugs are scarce, so the development of related new drugs is of great value and significance. However, on the whole, the total number of compounds entering clinical research in this field is small, the structural types are not sufficiently rich, and the IC_50_ values of these compounds that have entered the clinical stage are all several to tens of μM, and further improvement of the activity is needed. Therefore, with the help of computer-supported drug design methods that optimize the structural characteristics of target proteins and by focusing on natural product components and their structural modifications, the design and development of highly active and selective Nrf2 agonists will provide the possibility for the discovery of novel drug molecules in the future.

## Figures and Tables

**Table 1:** Seven Nrf2 agonists used in some clinical trials for COVID-19.

Intervention	Topic	Phase	Trial Country	Primary Endpoints	Dose	Subjects	Registration Number
EGCG **(1)**	Previfenon^®^ as Chemoprophylaxis of COVID-19 in Health Workers	2/3	Unknown	Event of clinical acute respiratory disease with a diagnosis of COVID-19 confirmed with rtPCR	250 mg/8 h orally for 40–70 days	Sample size: 524; Gender: all; Ages: 25 years and older.	NCT04446065
Sulforaphane **(2)**	CO-Sprout: Broccoli sprout powder for COVID-19 positive pregnant women on the rate of hospital admission	2	Australia	Duration of COVID-19 associated symptoms (days) as self-reported by trial participants.	21 mg, orally twice a day, morning and night (BD)	Sample size: 60; Gender: females; Ages: 18 years and older.	ACTRN12622000173796
	SFX-01 treatment for Acute Respiratory Infections (STAR-Covid19)	2	United Kingdom	1. Not hospitalized, no limitations on activities; 2. Not hospitalized, limitation on activities; 3. Hospitalized, not requiring supplemental oxygen; 4. Hospitalized, requiring supplemental oxygen; 5. Hospitalized, on non-invasive ventilation or high flow oxygen devices; 6. Hospitalized, on invasive mechanical ventilation or ECMO (Extracorporeal membrane oxygenation); 7. Death; timepoint(s) of evaluation of this end point day 15 (where day 1 is the first day of treatment).	300 mg, orally	Sample size: 300; Gender: all; Ages: adults (18–64 years): 120, elderly (≥65 years): 180.	EUCTR2020-003486-19-GB
Resveratrol **(3)**	The Anti-fibrotic Therapeutic Effects of Resveratrol for Discharged COVID-19 Patients	N/A	Hong Kong, China	1. The handheld basic spirometry; 2. PRO scores; 3. Borg Category Ratio 0–10 Scale;	1.0 g, orally once a day for six months.	Sample size: 30; Gender: all; Ages: 18 years to 65 years	NCT04799743
	Retrospective Study of ImmunoFormulation for COVID-19	N/A	Spain	Clinical symptoms duration. Time Frame: 1 month, starting after start of treatment.	transfer factors (oligo- and polypeptides from porcine spleen, ultrafiltered at <10 kDa—Imuno TF^®^) 100 mg, 800 mg anti-inflammatory natural blend (Uncaria tomentosa, Endopleura uchi and Haematoccocus pluvialis—MiodesinTM), 60 mg zinc orotate, 48 mg selenium yeast (equivalent to 96 μg of Se), 20,000 IU cholecalciferol, 300 mg ascorbic acid, 480 mg ferulic acid, 90 mg resveratrol, 800 mg spirulina, 560 mg N-acetylcysteine, 610 mg glucosamine sulphate potassium chloride, and 400 mg maltodextrin-stabilized orthosilicic acid (equivalent to 6 mg of Si—SiliciuMax^®^).	Sample size: 40; Gender: all; Ages: 18 years and older	NCT04666753
	Evaluation of the combined effect of Hesperidin, Artemisinin-Artemisia annua, Noscapine, N-acetylcysteine, Resveratrol supplements and high dose of vitamin C on treatment, clinical symptoms of non-hospitalization and hospitalization patients with symptomatic COVID-19	3	Iran (Islamic Republic of)	LDH, CBC diff, Na/K/Ca, CRP, ESR1, Weakness and nausea, respiratory quality	1. Artemisia annua—Artemisinin (manufactured by Longlifenutri)150 mg every 12 h; 2. Dose of 1 g of vitamin C (manufactured by Daroupakhsh) intravenous vitamin (two 500 mg ampoules in 250 cc of sodium chloride serum for 30 min) every 12 h; 3. Dose of 5 cc of noscapine (manufactured by Faran Shimi) every eight h; 4. 500 mg dose of hesperidin (manufactured by Swanson) every 24 h; 5. resveratrol 500 mg (Manufactured by A Squard) every 24 h; 6. NAC 600 mg (Manufactured by Osvah) every 12 h. The duration of treatment is estimated to be ten days. Supplements are taken orally, and vitamin C is given to patients as an injection.	Sample size: 100; Gender: all; Ages: 12 years and older	IRCT20181030041504N1
	Can SARS-CoV-2 Viral Load and COVID-19 Disease Severity be Reduced by Resveratrol-assisted Zinc Therapy	2; Terminated (Difficulty accruing patients)	United States	1. Reduction in SARS-CoV-2 viral load; 2. Reduction in severity of COVID-19 Disease.	Zinc Picolinate (50 mg po TID × 5 days); resveratrol (2 g po BID × 5 days)	Sample size: 45; Gender: all; Ages: 18 years to 75 years	NCT04542993
	Randomized Controlled Trial of Resveretrol-Copper OR Sodium-Copper-Chlorophyllin Versus Standard Treatment In Severe COVID-19 Cancer Patients	2	India	The time to clinical improvement, defined as a two-point improvement on a seven-point ordinal scale.	Tablet of resveratrol-Cu containing 5.6 mg of resveratrol and 560 ng of copper, orally once every 6 h.	Sample size: 200; Gender: all; Ages: 18 years to 99 years	CTRI/2020/07/026514
	Randomized Controlled Trial of Resveretrol-Copper Or Sodium-Copper-Chlorophyllin Vs Standard Treatment In Mild COVID-19 infection with Cancer Patients	3	India	The proportion of patients who suffer clinical deterioration OR viral persistence at Day 10 from the date of randomization (excluding the date of randomization).		Sample size: 300; Gender: all; Ages: 18 years to 99 years	CTRI/2020/07/026515
	Resveratrol and copper for the treatment of COVID-19 pnuemonia	N/A	India	To retrospectively access the clinical outcomes in the patients receiving R-Cu along with standard treatment versus those who received standard treatment.	N/A	Sample size: 230; Gender: all; Ages: 18 years to 99 years	CTRI/2020/06/026256
	Resveratrol in COVID-19	3	Iran (Islamic Republic of)	1. Time to clinical recovery; 2. Respiratory signs; 3. Intubation rate	500 mg, orally once a day for 14 days.	Sample size: 50; Gender: all; Ages: no age limit.	IRCT20200112046089N1
	Randomized Controlled Trial of Resveretrol-Copper Or Sodium-Copper-Chlorophyllin Vs Standard Treatment In Mild COVID-19 infection	3	India	1.Proportion of patients who suffer clinical deterioration OR viral infection; 2. Persistence at day 10 from the date of randomization (excluding the date of randomization); 3. Clinical deterioration will be defined as defined as a two-point or greater deterioration on a seven-point ordinal scale in every patient measured on each day, until day 10 from the date of randomization.	Tablet of resveratrol-Cu containing 5.6 mg of resveratrol and 560 ng of copper, orally once every 6 h.	Sample size: 300; Gender: all; Ages: 18 years to 99 years	CTRI/2020/05/025336
	Randomized Controlled Trial of Resveretrol-Copper OR Sodium-Copper-Chlorophyllin Versus Standard Treatment In Severe COVID-19	2	India	The time to clinical improvement, defined as a two-point improvement on a seven-point ordinal scale.		Sample size: 200; Gender: all; Ages: 18 years to 99 years	CTRI/2020/05/025337
	Evaluation efficacy of Curcumin and Resveratrol capsule in controlling symptoms in patients with COVID-19	3	Iran (Islamic Republic of)	Clinical symptoms changes (dry cough, respiratory distress, fever).	“Curcumin and Resveratrol” capsule (each capsule contains 200 mg of curcumin, 200 mg of resveratrol as active ingredients and 100 mg of lactose as filler), 1 capsule every 12 h for 7 days	Sample size: 60; Gender: all; Ages: 18 years and older.	IRCT20080901001165N56
	Randomized Proof-of-Concept Trial to Evaluate the Safety and Explore the Effectiveness of Resveratrol, a Plant Polyphenol, for COVID-19	2 (Terminated (Feasibility))	United States	Hospitalization rates for COVID-19: proportion of study participants admitted to the hospital within 21 days of randomization	Resveratrol 1000 mg 4 times/day for 15 days. Vitamin D3 100,000 IU on day 1	Sample size: 100; Gender: all; Ages: 45 years and older.	NCT04400890
Dimethyl Fumarate **(4)**	The Efficacy of dimethyl fumarate in the treatment of patients with COVID-19	2/3	Iran (Islamic Republic of)	Death; need for mechanical ventilation; severe illness.	240 mg capsules (CinnaGen, Tehran, Iran) daily for 5 days	Sample size: 30; Gender: all; Ages: 18 years and older.	IRCT20201024049134N4
	Randomised Evaluation of COVID-19 Therapy	2/3	United Kingdom; Nepal; Sri Lanka; Ghana; Vietnam; Indonesia; India; South Africa	All-cause mortality: For each pairwise comparison with the “no additional treatment” arm, the primary objective is to provide reliable estimates of the effect of study treatments on all-cause mortality.	120 mg every 12 h for 4 doses followed by 240 mg every 12 h by mouth for 8 days (10 days in total).	Sample size: 50,000; Gender: all; Ages: child, adult and older adult.	NCT04381936
Curcumin **(5)**	The Effect of Micellized Food Supplements on Health-related Quality of Life in Patients with Post-acute COVID-19 Syndrome.	N/A	Unknown	Change in health-related quality of life. Health-related quality of life is measured with the “Short-Form 12” (SF-12) from 0 to 100.	The daily intake of 2 × 10 drops of a mixture of micellized curcumin (2%), Boswellia serrata (1.5%) and ascorbic acid (6%).	Sample size: 32; Gender: all; Ages: 18 years to 85 years	NCT05150782
	Nutritional Supplementation of Flavonoids Quercetin and Curcumin for Early Mild Symptoms of COVID-19	N/A	Pakistan	1. Testing negative for SARS-CoV-2 using RT-PCR; 2. COVID-19 symptom improvement.	N/A	Sample size: 50; Gender: all; Ages: 18 years and older.	NCT05130671
	Determining the Safety and Effectiveness of ENDOR Oral Combination Drug in the Treatment of Patients with COVID-19	3	Iran (Islamic Republic of)	Clinical symptoms, radiological findings, laboratory findings.	two oral capsules of Endor every 8 h for 7 days. This capsule contains beta-carotene 2.5 mg; curcumin 23.75 mg; DHA 30 mg; EPA 45 mg; vitamin C 50 mg; wheat germ oil 75 mg; zinc 10 mg.	Sample size: 200; Gender: all; Ages: 18 years and older.	IRCT20100601004076N26
	Nanocurcumin (6C & 30C) on incidence of ILI & COVID-19 type respiratory illness	3	India	Incidence of influenza-like illness and COVID-19-type respiratory illness. Timepoint weekly until completion of 1 year.	Children below 5 years: 2 pills of medicine Nanocurcumin 6C once a week for first 2 months, followed by once in 2 weeks. Individuals 5 years or above: 4 pills of medicine Nanocurcumin 6C once a week for first 2 months, followed by once in 2 weeks.	Sample size: 17,000; Gender: all; Ages: 1 years and over.	CTRI/2021/08/035906
	Effect of Bromelain, Curcumin and Epigallocatechin in the treatment of outpatient COVID-19 patients	3	Iran (Islamic Republic of)	Blood oxygen saturation, sense of smell, sense of taste, fever, lung involvement, cough, muscle pain, weakness, gastrointestinal symptoms, death, hospitalization.	Each capsule contained 150 mg Bromelain, 300 mg Curcumin and 50 mg epigallocatechin; orally twice a day for 5 days	Sample size: 300; Gender: all; Ages: 18 years and over.	IRCT20210724051971N1
	Study Designed to Evaluate the Effect of CimetrA in Patients Diagnosed With COVID-19	2	Israel	1. Change in WHO Ordinal Scale for Clinical Improvement; 2. Change in COVID-19-Related Symptoms score; 3. Safety endpoint: will be assessed through collection and analysis of adverse events, blood and urine laboratory test, blood pressure and saturation, body temperature. Time frame: up to 28 days	CimetrA-1 containing a combination of curcumin 40 mg, Boswellia 30 mg and Vitamin C 120 mg. CimetrA-2 containing a combination of Curcumin 28 mg, Boswellia 21 mg and vitamin C 84 mg. Spray administration twice a day on days 1 and 2.	Sample size: 240; Gender: all; Ages: 18 years and over.	NCT05037162
	Clinical Study Designed to Evaluate the Effect of CimetrA in Patients Diagnosed With COVID-19	3	Israel	Clinical improvement in treatment groups. Time frame: up to 28 days. Time to sustained clinical improvement, defined as a national Early Warning Score 2 (NEWS2) of 2 maintained for 24 h in comparison to routine treatment (measured on days 7, 14, 28)	CimetrA-1 containing a combination of artemisinin 12 mg, curcumin 40 mg, Boswellia 30 mg, and vitamin C 120 mg. CimetrA-2 containing a combination of artemisinin 8.4 mg, curcumin 28 mg, Boswellia 21 mg, and Vitamin C 84 mg. Spray administration, twice a day on daays1 and 2.	Sample size: 252; Gender: all; Ages: 18 years and over.	NCT04802382
	A clinical study to see the effect of ArtemiC in patients with COVID-19	2	India	1.Time to clinical improvement, defined as a national Early Warning Score 2 (NEWS2); 2. Percentage of participants with definite or probable drug-related adverse events.	ArtemiC containing 12 mg artemisinin, 40 mg curcumin, 30 mg frankincense and 120 mg vitamin C as a maximum dose per 24 h, nasal spray, twice a day	Sample size: 20; Gender: all; Ages: 18 years to 65 years.	CTRI/2021/02/031520
	Oral Curcumin, Quercetin and Vitamin D3 Supplements for Mild to Moderate Symptoms of COVID-19	N/A	Pakistan	1.SARS-CoV-2 negativity determined by RT-PCR. Time frame: up to 14 days; 2. COVID-19 symptom improvement. Time frame: up to 7 days.	168 mg curcumin, 260 mg quercetin, and 360 IU of vitamin D3 orally once a day for 14 days.	Sample size: 50; Gender: all; Ages: 18 years and older.	NCT04603690
	Assessment of the effect of nanocurcumin supplement in patients with COVID-19	N/A	Iran (Islamic Republic of)	hs-CRP, recovery percentage, percentage of oxygen saturation, severity of infection symptoms of upper and lower respiratory tract, CBC.	80 mg nanocurcumin orally once every 12 h for 6 days.	Sample size: 48; Gender: all; Ages: 30 years to 70 years old.	IRCT20131125015536N13
	Effect of curcumin in treatment of respiratory syndrome of corona	2/3	Iran (Islamic Republic of)	Body temperature; oxygen saturation; chest CT-scan at the beginning of the study and on the third and seventh days.	150 mg curcumin orally every 8 h for 7 days.	Sample size: 42; Gender: all; Ages: no age limit.	IRCT20200418047119N1
	Evaluation of the effect of curcumin in improving patients with COVID-19	3	Iran (Islamic Republic of)	CT-scan findings; Hospitalization duration; CBC; LDH; PT; PTT; D-DIMER; BUN/CR.	Patients are given 3 curcumin capsules (500 mg) daily after three meals.	Sample size: 60; Gender: all; Ages: 18 years to 70 years old.	IRCT20200514047445N1
	Curcumin for COVID-19 Pre Exposure Prophylaxis	4	India	SARS-CoV-2 infection rate Using RT-PCR. Time Frame: up to 12 weeks.	Oral curcumin capsule 500 mg twice daily (morning, evening) for 12 weeks.	Sample size: 200; Gender: all; Ages: 18 years to 70 years old.	CTRI/2020/07/026820
	A clinical study to see effect of ArtemiC in patients with COVID-19	2	India	1.Time to clinical improvement, defined as a national Early Warning Score 2 (NEWS2); 2. Percentage of participants with definite or probable drug-related adverse events. Time frame: up to 15 days.	ArtemiC is an oromucosal medical spray composed ofartemisinin (6 mg/mL), curcumin (20 mg/mL), frankincense (15 mg/mL) and vitamin C (60 mg/mL); spray administration two times a day on days 1 and 2. Each dose contains 1 mL (10 puffs/pushes on the spray bottle), total daily dose 2 mL (20 puffs/pushes on the spray bottle). The total treatment is 40 puffs over two days.	Sample size: 50; Gender: all; Ages: 18 years to 65 years old.	CTRI/2020/07/026789
	Evaluation of the effect of nano micelles containing curcumin (Sina Ccurcumin) as a therapeutic supplement in patients with COVID-19	N/A	Iran (Islamic Republic of)	1.COVID-19 symptoms improvement; 2. Changes in immune cell balance. Frame: up to 2 weeks.	Oral 40 mg nanocurcumin capsules four times a day for 2 weeks.	Sample size: 40; Gender: all; Ages: 18 years to 75 years old.	IRCT20200611047735N1
	Evaluation the anti-inflammatory effects of curcumin in the treatment of patients with COVID-19	3	Iran (Islamic Republic of)	Cytokine gene expression, cytokine serum levels, clinical symptoms, laboratory findings.	240 mg nanocurcumin for 7 days at the same time with common therapeutic protocol category	Sample size: 60; Gender: all; Ages: 18 years to 65 years old.	IRCT20200519047510N1
	Evaluation efficacy of Curcumin and Resveratrol capsule in controlling symptoms in patients with COVID-19	3	Iran (Islamic Republic of)	Clinical symptoms changes (dry cough, respiratory distress, fever).	Each capsule contains 200 mg of curcumin, 200 mg of resveratrol as active ingredients, 1 capsule every 12 h for 7 days.	Sample size: 60; Gender: all; Ages: 18 years and over.	IRCT20080901001165N56
	Effect of curcumin-piperine in patients with coronavirus (COVID-19)	N/A	Iran (Islamic Republic of)	CT of the chest, body temperature, length of hospital stay, hs-CRP, ESR, ALT, AST, LDH, BUN, creatinine, CBC, blood oxidative stress indices (SOD, MDA, TAC), Albumin, Severity of the disease, severity and number of coughs.	Two curcumin-piperine capsules (500 mg curcumin + 5 mg piperine) will be given daily for 2 weeks after lunch and dinner.	Sample size: 100; Gender: all; Ages: 20 years to 75 years old.	IRCT20121216011763N46
	Evaluation of SinaCurcumin capsule efficacy as an supplement therapy for mild to moderate COVID-19 in Mashhad	3	Iran (Islamic Republic of)	Rates of treatment response and adverse drug reactions.	Nanocurcumin capsule 40 mg, two capsules twice daily for 2 weeks, then one capsule twice daily for 2 weeks.	Sample size: 60; Gender: all; Ages: 18 years to 65 years old.	IRCT20200408046990N1
	Effects of nano curcumin supplementation on the reduction of inflammation and mortality in patients with coronavirus 2019 admitted to ICU ward of imam Reza hospital in Tabriz	2/3	Iran (Islamic Republic of)	Gene expression rate; cytokine secretion rate; clinical observations; laboratory observations.	Oral 240 mg of nanocurcumin in 3 capsules of 80 mg daily.	Sample size: 86; Gender: all; Ages: 18 years to 80 years old.	IRCT20200324046851N1
Fluoxetine **(6)**	Use of a Combined Regimen of Fluoxetine, Prednisolone and Ivermectin in the Treatment of Mild COVID-19 to Prevent Disease Progression in Papua New Guinea	2/3	Papua New Guinea	COVID-19 disease progression (time frame: up to 14 days); SARS-CoV-2 viral load (time frame: up to 7 days)	Fluoxetine 20 mg oral tablets daily for 9 days. Prednisolone 25 mg oral tablets daily for 4 days. Ivermectin 3 mg oral tablets daily for 5 days.	Sample size: 954; Gender: all; Ages: 18 years to 99 years old.	NCT05283954
	efficacy and safety of adding fluoxetine to therapeutic regimen of patients with COVID-19 pneumonia	3	Iran (Islamic Republic of)	Blood oxygen saturation; number of days of hospitalization; need for intubation; ICU admission; death.	Oral fluoxetine capsules for 28 days, with 10 mg for the first 4 days followed by 20 mg for the rest of the 4-week period.	Sample size: 72; Gender: all; Ages: 16 years to 65 years old.	IRCT20200904048616N1
	Fluoxetine to Reduce Hospitalization From COVID-19 Infection (FloR COVID-19)	Early Phase 1; Withdrawn (study timeline is not feasible)	United States	Rate of hospitalization; physical symptoms assessed through daily checklist. Time frame: 8 weeks	Orally daily following: week 1: one pill (20 mg), week 2: two pills (40 mg), weeks 3–6: three pills (60 mg), week 7: two pills (40 mg), week 8: one pill (20 mg)	Sample size: 0.	NCT04570449
	Fluoxetine to Reduce Intubation and Death After COVID19 Infection	4	United States	Hospitalizations, intubation, death. Time frame: 2 months.	Oral 20 mg to 60 mg daily for 2 weeks to 2 months.	Sample size: 2000; Gender: all; Ages: 18 years and older.	NCT04377308
Bardoxolone Methyl **(7)**	BARCONA: A Study of Effects of Bardoxolone Methyl in Participants With SARS-Corona Virus-2 (COVID-19)	2	United States	Number of serious adverse events. Time frame: 29 days.	Oral 20 mg once a day for the duration of hospitalization (until recovery) with a maximum treatment duration of 29 days.	Sample size: 40; Gender: all; Ages: 18 years and older.	NCT04494646

**Table 2:** Two Nrf2 agonists used in some clinical trials for RA.

Intervention	Topic	Phase	Trial Country	Primary Endpoints	Dose	Subjects	Registration Number
Dimethyl Fumarate **(4)**	Efficacy and Safety Study of BG00012 with Methotrexate in Patients With Active Rheumatoid Arthritis	2	Australia	The primary objective is the proportion of subjects with ACR20 response in their RA at Week 12.	480 mg/day, oral and 720 mg/day, oral	Sample size: 153; Gender: all; Ages: 18 Years to 75 Years	NCT00810836
Curcumin **(5)**	Curcuma Longa L in Rheumatoid Arthritis	1; terminated (insufficient enrollment)	United States	Number of participants with adverse events as a measure of safety and tolerability.	4 250 mg curcumin capsules twice a day for one month	Sample size: 3; Gender: all; Ages: 18 Years and older.	NCT02543931
	Curcumin in Rheumatoid Arthritis	Early phase 1	United States	American College of Rheumatology 20%. Time frame: 4-month period.	4 capsules once a day for 2 weeks, and then the dose will be increased to 4 capsules twice a day beginning at week 3. Subjects will remain at this dose for an additional 13 weeks for a total 16 weeks. After 16 weeks, the same procedures will be repeated for another 16 weeks	Sample size: 40; Gender: all; Ages: 18 Years to 75 Years.	NCT00752154

**Table 3:** Four Nrf2 agonists used in some clinical trials for AD.

Intervention	Topic	Phase	Trial Country	Primary Endpoints	Dose	Subjects	Registration Number
EGCG **(1)**	Prevention of Cognitive Decline in ApoE4 Carriers with Subjective Cognitive Decline After EGCG and a Multimodal Intervention	N/A	Spain	Preclinical Alzheimer Cognitive Composite Plus exe-like score (ADCS-PACC-like).	Oral 532 mg/day (weight > 50 kg). Oral 266 mg/day (weight < 50 kg).	Sample size: 200; Gender: all; Ages: 60 years to 80 years old.	NCT03978052
	Sunphenon EGCg (Epigallocatechin-Gallate) in the Early Stage of Alzheimer’s Disease	2/3	Germany	ADAS-COG (Score 0–70) (baseline to treatment). Time frame: 18 months.	months 1–3: 200 mg/day (200-0-0 mg); months 4–6: 400 mg/day (200-0-200 mg); months 7–9: 600 mg/day (400-0–200 mg); months 10–18: 800 mg/day (400-0-400 mg)	Sample size: 21; Gender: all; Ages: 60 years and older.	NCT00951834
	Sunphenon EGCg (Epigallocatechin-Gallat) in the early stage of Alzheimer’s Disease—SUN-AK	2	Germany			Sample size: 50; Gender: all; Ages: 18 years and older.	EUCTR2009-009656-20-DE
Sulforaphane **(2)**	Effects of Sulforaphane in Patients with Prodromal to Mild Alzheimer’s Disease	N/A	China	The Alzheimer’s Disease Assessment Scale.	Oral 2550 mg once a day for 24 weeks.	Sample size: 160; Gender: all; Ages: 50 years to 75 years old.	NCT04213391
Resveratrol **(3)**	BDPP Treatment for Mild Cognitive Impairment (MCI) and Prediabetes or Type 2 Diabetes Mellitus (T2DM)	1	United States	Assessment of AEs and SAEs. Brain penetrance of BDPP. Neuropsychiatric Inventory and Cornell Scale for Depression in Dementia. Memory, executive function, and attention measures (composite).	N/A	Sample size: 14; Gender: all; Ages: 50 years to 90 years old.	NCT02502253
	Short Term Efficacy and Safety of Perispinal Administration of Etanercept in Mild to Moderate Alzheimer’s Disease	1	United States	Difference in effects of treatment for 6 weeks with etanercept + nutritional supplements versus nutritional supplements alone on the Mini-Mental Status Examination (MMSE) score.	N/A	Sample size: 12; Gender: all; Ages: 60 years to 85 years old.	NCT01716637
	Resveratrol for Alzheimer’s Disease	2	United States	Number of adverse events. Change from baseline in volumetric magnetic resonance imaging (MRI).	Begin at 500 mg taken once daily and increase after 13 weeks to 1 g taken by mouth twice daily.	Sample size: 119; Gender: all; Ages: 50 years and older.	NCT01504854
	Pilot Study of the Effects of Resveratrol Supplement in Mild-to-moderate Alzheimer’s Disease	3; withdrawn (PI has left institution)	United States	Cognition. Time frame: 52 weeks.	Oral 215 mg once a day for 52 weeks.	Sample size: 0.	NCT00743743
	Randomized Trial of a Nutritional Supplement in Alzheimer’s Disease	3	United States	Alzheimer Disease Assessment Scale (ADAScog). Time frame: one year.	N/A	Sample size: 27; Gender: all; Ages: 50 years to 90 years old.	NCT00678431
Curcumin **(5)**	KARVIAH_XTND: Longitudinal follow-up study examining the health and wellbeing of participants for identifying new biomarkers and the impact of lifestyle. (Following a 12 month intervention of curcumin for the prevention of Alzheimer’s disease.)	N/A	Australia	Blood biomarker compared with the brain amyloid levels. Blood biomarkers and PET imaging results.	N/A	Sample size: 100; Gender: all; Ages: 65 years and older.	ACTRN12620001325998
	Curcumin and Yoga Therapy for Those at Risk for Alzheimer’s Disease	2	United States	Curcumin effects (first six-month period) or curcumin and aerobic yoga effects (second six-month period) on the changes in the levels of blood biomarkers for mild cognitive impairment relative to baseline or relative to placebo or non-aerobic yoga.	Oral 800 mg curcumin in 4 capsules BID per day prior to meals.	Sample size: 80; Gender: all; Ages: 50 years to 90 years old.	NCT01811381
	KARVIAH Sub-study: Examining the use of curcumin on cognition and mood in an older population	2	Australia	Attention tasks and working memory as measured using a computerized cognitive battery (CogState).	Oral 500 mg 3 times daily.	Sample size: 40; Gender: all; Ages: 65 years to 90 years old.	ACTRN12616001113448
	Effect of curcumin (tumeric) in Alzheimer’s disease	N/A	Iran (Islamic Republic of)	MMSE and quality of life questionnaires. Time frame: before and after intervention (12 weeks).	Oral 500 mg twice a day for 12 weeks.	Sample size: 70; Gender: all; Ages: no age limit.	IRCT201507271165N11
	The epigenetic effect of curcumin as measured in the blood and seen within lifestyle, for the prevention of Alzheimer’s disease	2	Australia	Measurement of blood biomarkers within healthy and MCI groups.	Oral 1.5 mg daily (×3 divided doses) for a period of 3 or 6 months.	Sample size: 60; Gender: all; Ages: 65 years to 90 years old.	ACTRN12614001024639
	McCusker KARVIAH: Curcumin in Alzheimer’s disease prevention	2	Australia	AD-related blood biomarker profiles. Pib PET imaging. Neuropsychological tests. Time frame: up to 12 months.	500 mg daily for 2 weeks, progressing to 500 mg twice daily (1000 mg/daily) for 2 weeks, then 500 mg three times daily (1500 mg) for a period of 12 months in total.	Sample size: 134; Gender: all; Ages: 65 years to 90 years old.	ACTRN12613000681752
	Biocurcumax from curry spice turmeric in retaining cognitive function	N/A	Australia	Psychometric testing using Mini-Mental State Examination (MMSE), CAMDEX-R and (CAMCOG)-R, etc.	Oral 500 mg three times daily (total 1500 mg/day).	Sample size: 134; Gender: all; Ages: 65 years to 90 years old.	ACTRN12611000437965
	Efficacy and Safety of Curcumin Formulation in Alzheimer’s Disease	2	India	To determine if curcumin formulation affects mental capacity in Alzheimer’s patients based on mental exams.	Oral 2000 mg or 3000 mg daily BID.	Sample size: 26; Gender: all; Ages: 50 years to 80 years old.	NCT01001637
	A Pilot Study of Curcumin and Ginkgo for Treating Alzheimer’s Disease	1/2	Hong Kong, China	Change in isoprostane level in plasma. Change in A-beta level in serum.	Oral 1 g/4 g once daily.	Sample size: 36; Gender: all; Ages: 50 years and older.	NCT00164749
	Curcumin in Patients with Mild to Moderate Alzheimer’s Disease	2	United States	Side effect checklist.	N/A	Sample size: 33; Gender: all; Ages: 50 years and older.	NCT00099710

**Table 4:** Four Nrf2 agonists used in some clinical trials for PD.

Intervention	Topic	Phase	Trial Country	Primary Endpoints	Dose	Subjects	Registration Number
Vitamin D3 **(8)**	The Effects of Vitamin D and Bone Loss in Parkinson’s Disease	2	United States	Direct changes in bone formation and resorption will be investigated by measuring serum 25-hydroxyvitamin D [25(OH)D] level, serum parathyroid hormone (PTH) levels, serum osteocalcin, and serum n-telopeptides (N-Tx). Time frame: 12 months.	1000 IU/day of vitamin D3.	Sample size: 23; Gender: all; Ages: 18 years and older.	NCT00907972
	Clinical Effects of Vitamin D Repletion in Patients With Parkinson’s Disease	4	United State	Change from baseline visit to 3 months (treatment visit #1) in the TUG, timed walking task (8 m) and UPDRS III subscore. Time frame: 6 months.	600 IU vitamin D3 capsule daily.	Sample size: 31; Gender: All; Ages: 18 years to 89 years.	NCT00571285
	12 Weeks Vitamin D Supplementation and Physical Activity in PD Patients With DBS	Not Applicable	Poland	The effects of vitamin D supplementation and physical activity on concentration of vitamin D3 in serum—the evaluation of changes before and after 12 weeks of supplementation and physical activity. Time frame: the outcome will be assessed up to 1 year after the last collection of blood.	Dosage based on the BMI as followed: for BMI under 25—4000 IU/day, for BMI between 25 and 30—5000 IU/day, and for BMI over 30—6000 IU/day.	Sample size: 72; Gender: all; Ages: 40 years to 90 years.	NCT04768023
	Effects of Vitamin D in Parkinson’s Disease (PD)	2	United States	Change in static balance as recorded using dynamic posturography with the sensory organization test (SOT 1–3).	Drug: vitamin D3 Vitamin D3 at 10,000 IU a day. Dietary supplement: calcium 1000 mg calcium daily.	Sample size: 101; Gender: all; Ages: 50 years to 99 years.	NCT01119131
Resveratrol **(3)**	Tolerability, Safety and Pharmacokinetics of Four Single-doses of BIA 6-512 (Trans-resveratrol) and Their Effect on the Levodopa Pharmacokinetics	1	Portugal	1.Maximum observed plasma drug concentration (Cmax) post-dose—levodopa. Time of occurrence of Cmax (tmax)—levodopa; 2. Area under the plasma concentration–time curve (AUC) from time zero to the last sampling time at which concentrations were at or above the limit of quantification (AUC0-t), calculated by the linear trapezoidal rule—–evodopa; 3. Area under the plasma concentration versus time curve from time zero to infinity (AUC0-∞), calculated from AUC0-t + (Clast/λz), where Clast is the last quantifiable concentration and λz is the apparent terminal rate constant—levodopa; 4. Apparent terminal half-life, calculated from ln 2/λz (t1/2)—levodopa; 5. Maximum observed plasma drug concentration (Cmax) post-dose—BIA 6-512; 6. Time of occurrence of Cmax (tmax)—BIA 6-512; 7. Area under the plasma concentration–time curve (AUC) from time zero to the last sampling time at which concentrations were at or above the limit of quantification (AUC0-t), calculated by the linear trapezoidal rule—BIA 6-512. 8; Area under the plasma concentration versus time curve from time zero to infinity (AUC0-∞), calculated from AUC0-t + (Clast/λz), where Clast is the last quantifiable concentration and λz the apparent terminal rate constant—BIA 6-512; 9. Apparent terminal half-life calculated from ln 2/λz (t1/2)—BIA 6-512.	One capsule of Madopar^®^ HBS 125 (levodopa 100 mg/benserazide 25 mg) in an open label manner, concomitantly with BIA 6-512/Placebo.	Sample size: 20; Gender: all; Ages: 18 years to 45 years.	NCT03091543
	Effect of BIA 6-512 at Steady-state on the Levodopa Pharmacokinetics With a Single-dose of Levodopa/Benserazide 200/50 mg or With a Single-dose of Levodopa/Benserazide 200/50 mg Plus a Single-dose of Nebicapone 150 mg	1	Portugal	1.Day 4—Maximum observed plasma drug concentration (Cmax); 2. Day 4—Time of occurrence of Cmax (tmax); 3. Day 4—Area under the plasma concentration-time curve (AUC) from time zero to the last sampling time at which concentrations were at or above the limit of quantification (AUC0-t); 4. Day 4—AUC from time zero to 8 h post-dose (AUC0-τ); 5. Day 4—Area under the plasma concentration versus time curve from time zero to infinity (AUC0-∞); 6. Day 4—Apparent terminal elimination half-life, calculated from ln 2/λz (t1/2). 7; Day 5—Maximum observed plasma drug concentration (Cmax); 8. Day 5—Time of occurrence of Cmax (tmax); 9. Day 5—Area under the plasma concentration-time curve (AUC) from time zero to the last sampling time at which concentrations were at or above the limit of quantification (AUC0-t); 10. Day 5—AUC from time zero to 8 h post-dose (AUC0-τ); 11. Day 5—Area under the plasma concentration versus time curve from time zero to infinity (AUC0-∞); 12. Day 5—Apparent terminal elimination half-life, calculated from ln 2/λz (t1/2).	The investigational products consisted of capsules containing BIA 6-512 25 mg, 50 mg, 75 mg, 100 mg. Orally, with 240 mL of potable water.	Sample size: 38; Gender: all; Ages: 18 years to 45 years.	NCT03097211
EGCG **(1)**	Efficacy and Safety of Green Tea Polyphenol in De Novo Parkinson’s Disease Patients	2	China	Delay of progression of motor dysfunction.	N/A	Sample size: 480; Gender: all; Ages: 30 years and older.	NCT00461942
Sulforaphane **(2)**	A 6-month Study to Evaluate Sulforaphane Effects in PD Patients	2	China	Cognitive improvement assessed using the MATRICS Consensus Cognitive Battery (MCCB) composite score.	N/A	Sample size: 100; Gender: all; Ages: 40 years to 75 years.	NCT05084365

**Table 5:** Three Nrf2 agonists used in some clinical trials for SLE.

Intervention	Topic	Phase	Trial Country	Primary Endpoints	Dose	Subjects	Registration Number
Curcumin **(5)**	Effect of Curcumin on Systemic Lupus Erythematosus	2	California, United States	Change in SLEDAI.	Intervention is 2 g of curcumin supplement per day.	Sample size: 23; Gender: all; Ages: 18 years and older	NCT03953261
	Vitamin D and Curcumin Piperine Attenuates Disease Activity and Cytokine Levels in Systemic Lupus Erythematosus Patients	2	Indonesia	1.Disease activity from the SLE patients after the Treatments; 2. Fatigue assessment from the SLE patients after the treatments; 3. Comparison of cytokine levels before and after the treatments.	The third group received 400 IU cholecalciferol (Nature Plus) t.i.d and curcumin (600 mg)—piperine (15,800 mg) (Bioglan) one time daily.	Sample size: 45; Gender: all; Ages: 18 years to 45 years	NCT05430087
Vitamin D3 **(8)**	Vitamin D3 Treatment in Pediatric Systemic Lupus Erythematosus	2	California	Change in average IFN module expression level Percentage of Subjects by treatment arm experiencing any adverse event (AE) ≥ grade 3.	6000 IU of vitamin D3 by mouth daily until the subject’s serum 25 (OH) level is ≥ 40 ng/mL, at which point the supplementation dose is reduced to 4000 IU/day. Note: Subjects weighing < 40 kg (kg) at study entry will receive their dose five days a week and all other subjects seven days a week.	Sample size: 7; Gender: all; Ages: 5 years to 20 years.	NCT01709474
	Vitamin D3 in Systemic Lupus Erythematosus	2	United States	Percent of s with an IFN alpha signature response at Week 12.	8% vitamin D3 powder, 84% microcrystalline cellulose, 8% fumed silica by weight.	Sample size: 57; Gender: all; Ages: 18 years and older.	NCT00710021
	Vitamin D to Improve Endothelial Function in SLE	2	United States	Change at week 16 in % flow-mediated dilation in those who did and did not replete vitamin D.	5000 International units versus 400 international units as an active comparator.	Sample size: 9; Gender: all; Ages: 18 years and older.	NCT01911169
	Vitamin D Therapy in Patients With Systemic Lupus Erythematosus (SLE)	1	United States	Hypercalcuria.	Cholecalciferol 800 IU oral daily. Cholecalciferol 2000 IU oral daily. Cholecalciferol 4000 IU oral daily.	Sample size: 18; Gender: all; Ages: 18 years to 85 years.	NCT00418587
	Vitamin D and Curcumin Piperine Attenuates Disease Activity and Cytokine Levels in Systemic Lupus Erythematosus Patients	2	Indonesia	1. disease activity from the SLE patients after the treatments; 2. Fatigue assessment from the SLE patients after the treatments; 3. Comparison of cytokine levels before and after the treatment	The second group received a tablet containing curcumin (632 mg)—piperine (15,800 mg) (Bioglan) one time daily and a placebo (Saccharum lactis) t.i.d.	Sample size: 45; Gender: all; Ages:18 years to 45 years.	NCT05430087
	Effect of Vitamin D Supplement on Disease Activity in SLE	Not Applicable	Thailand	To examine the effect of vitamin D supplementation on SLE disease activity.	Add on vitamin D2 (calciferol) 40,000 IU/wk (2 cap) for 12 weeks.	Sample size: 100; Gender: all; Ages: 18 years and older.	NCT05260255
	The Effect of Vitamin D Supplementation on Disease Activity Markers in Systemic Lupus Erythematosus (SLE)	Not Applicable	Egypt	Decrease in SLE disease activity.	2000 IU/day for 12 months.	Sample size: 248; Gender: all; Ages: 30 years and older.	NCT01425775
SM934 **(9)**	Safety and Efficacy of SM934 Compared to Placebo in Adult Subjects With Active Systemic Lupus Erythematosus	2	China	1.Percentage of subjects with lupus low disease activity score (LLDAS) in each group; 2. Percentage of subjects with systemic lupus erythematosus responder index—4 (SRI-4) response in each group; 3. Percentage of subjects with treatment-emergent adverse events (TEAEs) in each group.	SM934 10 mg (5 tablet) p.o. qd in combination with steroids.	Sample size: 48; Gender: all; Ages: 30 years and older.	NCT03951259

**Table 6:** Three Nrf2 agonists used in some clinical trials for NAFLD.

Intervention	Topic	Phase	Trial Country	Primary Endpoints	Dose	Subjects	Registration Number
Liraglutide	Efficacy Study of Liraglutide vs. Sitagliptin vs. Glargine on Liver Fat in T2DM Subjects	4	China	To compare the change of intrahepatic lipids (IHL) in type 2 diabetic patients with nonalcoholic fatty liver disease after a 26-week treatment of liraglutide, sitagliptin, or insulin glargine per day combined with metformin.	Liraglutide, 0.6 mg per day for the first week, increased to 1.2 mg per day for the second week, and finally 1.8 mg per day from the third week.	Sample size: 75; Gender: all; Ages: 30 years to 75 years.	NCT02147925
	Antidiabetic Effects on Intrahepatic Fat	4	China	Intrahepatic fat change from baseline by quantitative ultrasound.	0.6 mg/day during the first week, 1.2 mg/day during the second week, and 1.8 mg/day from the third week.	Sample size: 87; Gender: all; Ages: 17 years to 80 years.	NCT03068065
	Liraglutide Efficacy and Action in Non-Alcoholic Steatohepatitis	2	England	Liver histological improvement.	1.8 mg once daily, subcutaneous injection.	Sample size: 52; Gender: all; Ages: 18 years to 70 years.	NCT01237119
	Study of Liraglutide Versus Insulin on Liver Fat Fraction in Patients With Type 2 Diabetes	2	Canada	Improvement in liver steatosis defined by change in liver fat fraction as measured by MRI and MR spectroscopy at baseline and 12 weeks of treatment.	0.6–1.8 mg subcutaneous per day.	Sample size: 35; Gender: all; Ages: 18 years and older.	NCT01399645
Resveratrol **(3)**	Long-term Investigation of Resveratrol on Fat Metabolism in Obese Men With Nonalcoholic Fatty Liver Disease	N/A	Denmark	Hepatic VLDL-TG secretion and peripheral VLDL-TG clearance. Time frame: six months. - Changes from baseline after treatment with either resveratrol or placebo.	500 mg 3 times daily for six months.	Sample size: 26; Gender: all; Ages: 25 years to 65 years.	NCT01446276
	Resveratrol for the Treatment of Non Alcoholic Fatty Liver Disease and Insulin Resistance in Overweight Adolescents	2/3	Canada	Primary Side effect profile determined by interview and serum biochemistry. Side effect profile determined by serum biochemistry: AST, ALT, total and conjugated bilirubin, Creatinine, sodium, potassium, calcium, magnesium, chloride and TC02, haemoglobin, haematocrit, white blood cell and platelet counts, erythrocytes, and fasting lipid levels (total cholesterol, HDL-cholesterol, LDL-cholesterol and triglycerides). Fasting glucose and insulin levels. PT/INR and PTT levels.	Oral 75 mg twice daily (with breakfast and dinner) for a total daily dose of 150 mg for the duration of 30 days.	Sample size: 10; Gender: all; Ages: 13 years to 18 years.	NCT02216552
	Resveratrol in Patients With Non-alcoholic Fatty Liver Disease	2/3	Denmark	Changes in hepatic and inflammatory markers ind the blood such as ALT, hs-CRP, TNFa; changes in hepatic fat content, assessed by MR spectroscopy; changes in hepatic steatosis and inflammation, assessed histologically; changes in the expression of proteins in the relevant inflammatory pathways, assessed by gene expression studies.	500 mg 3 times daily for 6 months.	Sample size: 28; Gender: all; Ages: 18 years to70 years.	NCT01464801
	The Effects of Resveratrol Supplement on Biochemical Factors and Hepatic Fibrosis in Patients With Nonalcoholic Steatohepatitis	2/3	America	Alaninaminotransferase (ALT).	One resveratrol capsule per day for 12 weeks.	Sample size: 50; Gender: all; Ages: 18 years to 80 years.	NCT02030977
	Potential Beneficial Effects of Resveratrol	N/A	Denmark	Metabolic parameters. Time frame: five weeks. Regarding glucose, protein, and fat metabolism.	500 mg three times a day for five weeks.	Sample size: 24; Gender: male; Ages: 18 years and older.	NCT01150955
Curcumin **(5)**	Curcumin for Pediatric Nonalcoholic Fatty Liver Disease	2	America	Change in serum alanine aminotransferase (ALT) from baseline. Time frame: 24 weeks. ALT value in U/L	500 mg daily phosphatidylcholine–curcumin complex supplement orally for 24 weeks.	Sample size: 0; Gender: all; Ages: 8 years to 17 years.	NCT04109742
	Curcumin Supplement in Nonalcoholic Fatty Liver Patients	2/3	America	Hepatic steatosis (time frame: 12 weeks) measured by CAP score using Fibroscan.	1500 mg one capsule/day for 12 weeks.	Sample size: 50; Gender: all; Ages: 18 years and older.	NCT02908152
	The Effect of Curcumin on Liver Fat Content in Obese Subjects	N/A	Denmark	Curcumin’s effect on steatosis. Time frame: 42 days ± 3 days. Percentage of fat in the liver tissue measured by magnetic resonance spectroscopy.	500 mg tablet (contains 100 mg curcumin); Dosage: 2 tablets twice daily for 42 days (± 3 days).	Sample size: 39; Gender: male; Ages: 20 years and older.	NCT03864783
	Efficacy of a Natural Components Mixture in the Treatment of Non Alcoholic Fatty Liver Disease (NAFLD)	N/A	Italy	Hematic levels of hepatic enzymes AST; hematic levels of hepatic enzymes ALT; hematic levels of hepatic enzymes GGT. Time frame: before and at the end of treatment (three months).	Nutraceutical mixture (two soft 800 mg gelatin capsules per day) for three months.	Sample size:126; Gender: male; Ages: 18 years to 80 years.	NCT02369536

**Table 7:** Silymarin used in some clinical trials for HCV.

Intervention	Topic	Phase	Trial Country	Primary Endpoints	Dose	Subjects	Registration Number
Silymarin **(10)**	Effects of Silybum Marianum on Treatment of Patients With Chronic Hepatitis C	2	Iran	The investigators measured serum amino transferases using commercial AST kits and ALT kits (Bayer Diagnostics, Tarrytown, NY, USA) at six months after silymarin admission.	210 mg tabs; 630 mg daily for six months.	Sample size: 55; Gender: all; Ages: child, adult, older adult.	NCT01292161
	Clinical Study With Silymarin in the Patients With Chronic Hepatitis C Infection Who Failed Conventional Antiviral Therapy	3	Korea	The proportion of patient with serum ALT less than or equal to 40 IU/L or achieves at least 50% decline to less than 60 IU/L.	700 mg thrice daily.	Sample size: 53; Gender: all; Ages: 18 years and older.	NCT01258686
	Phase II Trial of Silymarin for Patients With Chronic Hepatitis C Who Have Failed Conventional Antiviral Treatment	2	America	1. Efficacy—whether or not serum ALT (mg/dl) is less than or equal to 45 IU/L (approximate normal range) or achieves at least 50% decline to less than 65 IU/L (approximately 1.5 times the upper limit of normal); 2. Safety—occurrence of a dose-limiting toxicity. Time frame: 24-week treatment period.	1. 700 mg dose (5 pills, three times daily) for 24-week treatment period. 2. 420 mg dose (5 pills, three times daily) for 24-week treatment period.	Sample size: 154; Gender: all; Ages: 18 years and older.	NCT00680342
	Evaluating Silymarin for Chronic Hepatitis C	2	America	N/A	N/A	N/A	NCT00030030
	Randomized Placebo-controlled Trial Evaluating the Safety and Efficacy of Silymarin Treatment in Patients With Acute Viral Hepatitis	2/3	Egypt	1. Incidence, severity, and duration of adverse events. Time frame: four weeks after enrollment. 2. Normalization of total (< 1.0 mg/dl) and direct bilirubin (< 0.3 mg/dl). Time frame: four weeks after enrollment.	280 mg three times daily for four weeks.	Sample size: 199; Gender: all; Ages: 18 years and older.	NCT00755950
	Phase I Trial of Silymarin for Chronic Liver Diseases	1	America	Adverse events. Time frame: 10 days.	280 mg every 8 h.	Sample size: 56; Gender: all; Ages: 18 years and older.	NCT00389376
	Effect of LEGALON SIL on Hepatitis C Virus Recurrence in Stable Liver Transplanted Patients	2	Italy	To determine the effect of post-transplant treatment with Legalon SIL on HCV viral load 30 days after the beginning of treatment.	20 mg/kg silibinin, administered daily as a 2-h infusion for 14 days.	Sample size: 20; Gender: all; Ages: 18 years to 70 years.	NCT01518933

**Table 8:** Ursodeoxycholic Acid used in some clinical trials for PBC.

Intervention	Topic	Phase	Trial Country	Primary Endpoints	Dose	Subjects	Registration Number
Ursodeoxycholic Acid **(11)**	Efficacy and Safety Study of TUDCA Compare UDCA to Treatment Chronic Cholestatic Liver Disease -PBC	3	China	Efficiency is defined as the proportion of patients whose ALP levels of serum decreased more than 25% compared to baseline at treatment for 24 weeks.	250 mg/8 h orally for 24 weeks.	Sample size: 199; Gender: all; Ages: 18 years to 70 years.	NCT01829698
	Clinical Research of UCDA Reducing Medication Regimen in Stable PBC	4	China	Liver biochemical markers (AST and ALP in U/L, BIL in umol/L) that restored to normal increase (bilirubin > 17 μmol/L, ALP > 3 ULM, AST > 2 ULN) again are considered to be PBC recurrence. The rate of recurrence will be described in percent.	1. 250 mg orally twice a day; 2. 250 mg orally once a day; 3. 250 mg orally three times a day.	Sample size: 90; Gender: all; Ages: 18 years to 65 years.	NCT04650243
	Ursodeoxycholic Acid Combined With Low Dose Glucocorticoid in the Treatment of PBC With AIH Features II	4	China	The percentage of patients in biochemical remission, defined as normalization of serum ALT and IgG levels after treatment, per treatment group.	13–15 mg/kg/d.	Sample size: 90; Gender: all; Ages: 18 years to 70 years.	NCT04617561
	Ursofalk Tablets (500 mg) Versus Ursofalk Capsules (250 mg) in the Treatment of Primary Biliary Cirrhosis	4	Germany	Change of liver enzymes between baseline and the end of the treatment period with 250 mg Ursofalk capsules and the end of treatment period with 500 mg Ursofalk tablets.	500 mg orally for 24 weeks.	Sample size: 65; Gender: all; Ages: 18 years and older.	NCT01510860
	Development of Ursodeoxycholic Acid 300 mg at Hospital Das Clinicas of the University of São Paulo School of Medicine	N/A	America	Compare the liver enzyme parameters (alkaline phosphatase, alanine aminotranferase, aspartate. aminotransferase, gamma glutamyl transferase, and total bilirubin) in three different moments before the treatment, under the treatment, and at the end of treatment.	300 mg 13–15 mg/kg day for 3 months.	Sample size: 30; Gender: all; Ages: 18 years and older.	NCT03489889

**Table 9:** Candesartan used in clinical trials for liver fibrosis.

Intervention	Topic	Phase	Trial Country	Primary Endpoints	Dose	Subjects	Registration Number
Candesartan **(12)**	Effect of Some Drugs on Liver Fibrosis	3	Egypt	Change in Fibroscan or APRI score.	8 mg/day for 6 months.	Sample size: 45; Gender: all; Ages: 20 years and older.	NCT03770936

## Abbreviations

ACE2	angiotensin-converting enzyme 2
AD	Alzheimer’s disease
AIH	autoimmune hepatitis
AILI	APAP-induced liver injury
ALD	alcoholic liver disease
ALT	alanine aminotransferase
AMPK	adenosine 5‘-monophosphate (AMP)-activated protein kinase
Ang II	angiotensin II
APAP	acetaminophen
APP/PS1	amyloid precursor protein plus presenilin-1
ARE	antioxidant response element
ASH	alcoholic steatohepatitis
AST	aspartate aminotransferase
ATP	adenosine triphosphate
Aβ	β-amyloid
BACE	β-site amyloid-precursor-cleaving enzyme
BMDCs	bone-marrow-derived dendritic cells
CAT	catalase
cGMP	cyclic guanosine 3′, 5′-monophosphate
CIA	collagen-induced arthritis
CNS	central nervous system
COVID-19	Coronavirus Disease 2019
COX-2	Cyclooxygenase-2
Cul3	CULLIN3
CYP 450	cytochrome P450
CYP2E1	cytochrome P450 2E1
DAPI	4′,6-diamidino-2-phenylindole
DFT	density functional theory
DILI	drug-induced liver injury
DMF	Dimethyl fumarate
DPPH	2,2-diphenyl-1-picrylhydrazyl
dTHP-1	Human THP-1 cells differentiated to the macrophage phenotype
EC	Epieatechin
ECG	Epieatechin gallate
EGC	Epigalloeatechin
EGCG	(−)-epigallocatechin-3-gallate
FDA	Food and Drug Administration
GCL	glutamate cysteine ligase
GSH-Px	glutathione peroxidase
GST	glutathione-S-transferases
IC50	half maximal inhibitory concentration
IDMF	di-(methyl fumarate)
IFN-I	I interferons
IL-17	interleukin-17
IL-1β	interleukin 1 beta
IL-6	interleukin 6
iNOS	inducible nitric oxide synthase
GCLC	catalytic subunit of glutamate–cysteine ligase
GCLM	gutamate-cysteine ligase modifier subunitl
GSH	glutathione
GSK-3	glycogen synthase kinase 3
HAS	hydroxy-α-sanshool
HBV	hepatitis B
HBx	hepatitis B x protein
HCC	hepatocellular carcinoma
HCV	hepatitis C
HFD	high-fat diet
HMOX-1	heme-oxigenase-1
HO-1	heme-oxigenase-1heme-oxigenase-1
HSC	hepatic stellate cell ICC intrahepatic cholangiocarcinoma
Keap1	Kelch-like ECH-associated protein 1
LN	lupus nephritis
LPS	Lipopolysaccharide
LSEC	liver sinusoidal endothelial cell
MAF	musculoaponeurotic fibrosarcoma
MAO	Monoamine oxidase
MS	multiple sclerosis
NAFLD	non-alcoholic fatty liver disease
NAPQI	N-acetyl-1,4-benzoquinone imine
NDP52	nuclear dot protein 52
NF-κB	nuclear factor kappa B
NLR	nucleotide-binding domain leucine-rich repeat-related
NLRP3	nucleotide-binding domain leucine-rich repeat-related (NLR) family pyrin do-main-containing 3
NOX	NADPH-oxidase
NQO1	NAD (P) H-quinone oxidoreductase 1
Nrf2	nuclear factor E2-related factor 2
NS3	nonstructural protein 3
NS5A	nonstructural protein 5A
OSI	oxidative stress index
PBC	primary biliary cholangitis PSC primary sclerosing cholangitis
PD	Parkinson’s disease
PTEN	phosphatase and tensin homolog
PINK	phosphatase and tensin homolog (PTEN)-induced kinase
PSC	primary sclerosing cholangitis
qPCR	Quantitative Real-time PCR
RNA	Ribonucleic Acid
RA	Rheumatoid arthritis
ROS	reactive oxygen species
SARS-CoV-2	severe acute respiratory syndrome coronavirus 2
SFN	Sulforaphane
SLE	systemic lupus erythematosus
Socs3	suppresses cytokine signaling 3
SOD	superoxide dismutase
STAT3	Signal transducer and activator of transcription 3
TAS	total antioxidant status
Th17	T helper type 17
TLR7	Toll-like receptor 7
TLR9	Toll-like receptor 9
TMPRSS2	transmembrane protease serine 2
TNF-α	tumor necrosis factor alpha
TrX-1	thioredoxin-1
TRX	thioredoxin
TrxR1	thioredoxin reductase 1
TOS	total oxidant status
UDCA	ursodeoxycholic acid
α-syn	α-synuclein
γ-GCS	γ-glutamyl cysteine synthetase
